# Effects of women’s groups practising participatory learning and action on preventive and care-seeking behaviours to reduce neonatal mortality: A meta-analysis of cluster-randomised trials

**DOI:** 10.1371/journal.pmed.1002467

**Published:** 2017-12-05

**Authors:** Nadine Seward, Melissa Neuman, Tim Colbourn, David Osrin, Sonia Lewycka, Kishwar Azad, Anthony Costello, Sushmita Das, Edward Fottrell, Abdul Kuddus, Dharma Manandhar, Nirmala Nair, Bejoy Nambiar, Neena Shah More, Tambosi Phiri, Prasanta Tripathy, Audrey Prost

**Affiliations:** 1 Institute for Global Health, University College London, London, United Kingdom; 2 Department of Medical Statistics, London School of Hygiene & Tropical Medicine, London, United Kingdom; 3 Department of Infectious Disease Epidemiology, London School of Hygiene & Tropical Medicine, London, United Kingdom; 4 Nuffield Department of Medicine, Centre for Tropical Medicine, University of Oxford, Oxford, United Kingdom; 5 Perinatal Care Project, Diabetic Association of Bangladesh, Dhaka, Bangladesh; 6 Society for Nutrition, Education & Health Action, Mumbai, India; 7 Mother and Infant Research Activities (MIRA), Kathmandu, Nepal; 8 Ekjut, Chakradharpur, India; 9 Parent and Child Health Initiative Trust, Lilongwe, Malawi; London School of Hygiene & Tropical Medicine, UNITED KINGDOM

## Abstract

**Background:**

The World Health Organization recommends participatory learning and action (PLA) in women’s groups to improve maternal and newborn health, particularly in rural settings with low access to health services. There have been calls to understand the pathways through which this community intervention may affect neonatal mortality. We examined the effect of women’s groups on key antenatal, delivery, and postnatal behaviours in order to understand pathways to mortality reduction.

**Methods and findings:**

We conducted a meta-analysis using data from 7 cluster-randomised controlled trials that took place between 2001 and 2012 in rural India (2 trials), urban India (1 trial), rural Bangladesh (2 trials), rural Nepal (1 trial), and rural Malawi (1 trial), with the number of participants ranging between 6,125 and 29,901 live births. Behavioural outcomes included appropriate antenatal care, facility delivery, use of a safe delivery kit, hand washing by the birth attendant prior to delivery, use of a sterilised instrument to cut the umbilical cord, immediate wrapping of the newborn after delivery, delayed bathing of the newborn, early initiation of breastfeeding, and exclusive breastfeeding. We used 2-stage meta-analysis techniques to estimate the effect of the women’s group intervention on behavioural outcomes. In the first stage, we used random effects models with individual patient data to assess the effect of groups on outcomes separately for the different trials. In the second stage of the meta-analysis, random effects models were applied using summary-level estimates calculated in the first stage of the analysis. To determine whether behaviour change was related to group attendance, we used random effects models to assess associations between outcomes and the following categories of group attendance and allocation: women attending a group and allocated to the intervention arm; women not attending a group but allocated to the intervention arm; and women allocated to the control arm. Overall, women’s groups practising PLA improved behaviours during and after home deliveries, including the use of safe delivery kits (odds ratio [OR] 2.92, 95% CI 2.02–4.22; *I*^2^ = 63.7%, 95% CI 4.4%–86.2%), use of a sterile blade to cut the umbilical cord (1.88, 1.25–2.82; 67.6%, 16.1%–87.5%), birth attendant washing hands prior to delivery (1.87, 1.19–2.95; 79%, 53.8%–90.4%), delayed bathing of the newborn for at least 24 hours (1.47, 1.09–1.99; 68.0%, 29.2%–85.6%), and wrapping the newborn within 10 minutes of delivery (1.27, 1.02–1.60; 0.0%, 0%–79.2%). Effects were partly dependent on the proportion of pregnant women attending groups. We did not find evidence of effects on uptake of antenatal care (OR 1.03, 95% CI 0.77–1.38; *I*^2^ = 86.3%, 95% CI 73.8%–92.8%), facility delivery (1.02, 0.93–1.12; 21.4%, 0%–65.8%), initiating breastfeeding within 1 hour (1.08, 0.85–1.39; 76.6%, 50.9%–88.8%), or exclusive breastfeeding for 6 weeks after delivery (1.18, 0.93–1.48; 72.9%, 37.8%–88.2%). The main limitation of our analysis is the high degree of heterogeneity for effects on most behaviours, possibly due to the limited number of trials involving women’s groups and context-specific effects.

**Conclusions:**

This meta-analysis suggests that women’s groups practising PLA improve key behaviours on the pathway to neonatal mortality, with the strongest evidence for home care behaviours and practices during home deliveries. A lack of consistency in improved behaviours across all trials may reflect differences in local priorities, capabilities, and the responsiveness of health services. Future research could address the mechanisms behind how PLA improves survival, in order to adapt this method to improve maternal and newborn health in different contexts, as well as improve other outcomes across the continuum of care for women, children, and adolescents.

## Abstract 2

In a meta-analysis of randomized controlled trials, Nadine Seward and colleagues find that in Asian and African settings with limited access to medical services, participatory learning women’s groups are associated with improved perinatal health behaviors.

## Author summary

**Why was this study done?:**

- A systematic review and meta-analysis of trials of participatory learning and action in women’s groups found a 25% reduction in neonatal mortality associated with these groups, but the pathways to improved survival have not been explored using available evidence from all trials.
- We used data from cluster-randomised trials of women’s groups to explore behaviours in the antenatal, delivery, and postnatal periods in order to better explain the reduction in neonatal mortality associated with these groups. We also examined whether women who were assigned to the intervention arm and attended group meetings were more likely to have improved care practices than women who were also in the intervention arm but did not attend group meetings.

**What did the researchers do and find?:**

- We conducted a meta-analysis using individual-level data to explore the relationship between women’s groups and key behaviours in the antenatal, delivery, and postnatal periods. Our findings suggest that women’s groups are able to improve key behaviours for home deliveries including clean delivery practices and thermal care practices.
- To determine whether women who attended group meetings were more likely to have improved behaviours compared with women who did not attend, we compared behaviours between these women separately for the different trials. Overall, we found that women who attended group meetings were more likely to have improved behaviours than women who did not attend.

**What do these findings mean?:**

- Our meta-analysis showed that women’s groups were associated with improvements in critical practices including clean deliveries and appropriate thermal care for home deliveries. Evidence suggests that these care practices are essential for reducing neonatal mortality because of the importance of sepsis and hypothermia in areas with high neonatal mortality and low rates of facility births. Although this finding explains how women’s groups improved survival in these contexts, we also found that women’s groups improved survival in areas with lower neonatal mortality, such as rural Bangladesh and rural Malawi. It is possible that women’s groups were able to help families make more timely, better informed decisions about care seeking.
- Women’s groups have demonstrated flexibility in adapting to a shifting environment to improve birth outcomes through important pathways. Key to the continued reduction in adverse birth outcomes will be sustained improvement in community-level practices, as well as ensuring that health facilities are equipped to support quality care.

## Introduction

Between 1990 and 2015, mortality rates in children aged between 2 months and 5 years declined globally by 58% [1–3]. Neonatal mortality decreased by 47% over the same period, but the proportion of deaths occurring during the neonatal period out of all deaths among children under 5 years of age increased from 37% to 45% [3]. If these trends continue, neonatal mortality will constitute over 50% of deaths among children under 5 years of age by 2030 [3]. Increased coverage of effective interventions is required to improve neonatal survival [4].

Scaling up community interventions to improve maternal and newborn health outcomes has the potential to reduce neonatal mortality by 25% (risk ratio 0.75, 95% CI 0.67–0.83; 21 studies, *n* = 302,464). The most effective interventions are community mobilisation through women’s groups, counselling for care and referral through home visits, and combinations of these 2 approaches [5]. A meta-analysis of home visiting programmes with or without home-based neonatal care found that interventions in proof-of-principle studies led to a 45% reduction in neonatal mortality (relative risk 0.55, 95% CI 0.48–0.63), while interventions tested at scale, in programmatic conditions, led to a 12% reduction (risk ratio 0.88, 95% CI 0.82–0.95) [6]. A meta-analysis of 7 trials evaluating the effects of women’s groups practising participatory learning and action (PLA) found a 20% reduction in neonatal mortality (odds ratio [OR] 0.80; 95% CI 0.67–0.96) with high levels of heterogeneity (*I*^2^ = 73.2%, *p* = 0.001) [7]. The WHO and UNICEF Every Newborn Action Plan now recommends both home visits and participatory meetings with women’s groups as community strategies to improve maternal and newborn health [8].

In most of the studies included in the above-mentioned meta-analysis, women’s groups went through a PLA cycle with 4 distinct phases [7]. In the first phase, groups identified and prioritised common maternal and newborn health problems in their community. In the second phase, they discussed potential solutions and prioritised them. In the third phase, groups implemented their chosen solutions, and in the fourth, they evaluated their progress and planned for the future [7,9–13]. The cycle of meetings was intended to build the capacity of individuals, groups, and communities to take action to improve maternal and neonatal health [14].

Although women’s groups practising PLA have been shown to reduce newborn mortality in some settings, questions remain about the mechanisms through which they achieve this [7]. In rural eastern India, the proof-of-principle Ekjut cluster-randomised controlled trial and its process evaluation suggested that improved clean delivery practices and thermal care were partially responsible for increased neonatal survival [15]. In Malawi, the MaiMwana trial process evaluation noted that groups used varied strategies to address maternal and neonatal health concerns, including health education, bicycle ambulances, distribution of insecticide-treated nets, establishment of mobile antenatal and under-5 clinics, and group funds [14]. In Nepal, the process evaluation suggested that improvement in mortality was possibly due to increases in care-seeking and preventive care practices for home deliveries [16].

Results from the meta-analysis showing the value of women’s groups in improving neonatal survival were heterogeneous [7]. Although most of the trials in rural South Asia found reductions in neonatal mortality, this was not the case for the trial that took place in an urban Indian setting [7,17]. These findings and ongoing changes in the coverage of key strategies to improve maternal and neonatal survival, including facility-based deliveries, suggest a need to gain better insight into the mechanisms through which this complex intervention works.

We sought to examine the effects of women’s groups practising PLA on behaviours in the antenatal, delivery, and postnatal periods in order to understand the pathways to mortality reduction. Because the effects on neonatal mortality appeared to be greater in studies where more pregnant women attended meetings, we hypothesized that improved behaviours would also be related to whether a woman attended women’s group meetings [7].

## Methods

### Ethics

Ethical approval for the trials that collected the data for this study came from the UCL Great Ormond Street Institute of Child Health and Great Ormond Street Hospital for Children (UK) and in-country research ethics committees, as previously detailed [7].

### Search criteria

We did a meta-analysis of trials of women’s groups practising PLA. Our search strategy and inclusion criteria were similar to those of a previous systematic review and meta-analysis. Briefly, we searched PubMed, Embase, Cochrane Library, CINAHL, African Index Medicus, Web of Science, the WHO Reproductive Health Library, and the Science Citation Index for studies published from the databases’ inception dates until March 1, 2017, with no language restrictions. Search terms included a combination of ‘community mobilisation’, ‘community participation’, ‘participatory learning and action’, ‘women’s groups’, and ‘women’. We also sought unpublished data from researchers known to be active in this area. Studies were included if they were randomised controlled trials, participants were women aged 15–49 years, and the trial tested a PLA cycle with women’s groups and reported information on at least 1 of our chosen outcomes [7]. Six of the 7 studies in the previous review met our inclusion criteria, as did 1 additional study from rural India [13]. In total, our analysis included 7 trials that took place between 2001 and 2012 within socio-economically disadvantaged communities in 4 countries, including rural communities in Bangladesh, Malawi, and Nepal, and rural and urban communities in India [7,10–13,17–19]. We used individual-level data collected during these 7 cluster-randomised controlled trials.

### Included studies

Table 1 describes the characteristics of each study, including the number of participants. Two of the trials used a 2-by-2 factorial design. The first Bangladesh trial used a factorial design to assess the effects of the women’s group intervention and of a traditional birth attendant (TBA) training intervention [11]. There was no evidence of interaction between these 2 interventions, so we included data collected from all study participants [11]. The trial in Malawi used a factorial design to assess both the women’s group intervention and an infant feeding intervention. Because there was significant interaction between the 2 interventions and the infant feeding intervention had an independent effect on neonatal mortality, we did not include participants in the infant feeding arm in this analysis [20].

**Table 1:** Characteristics of trials of women’s group interventions included in this analysis.

Study	Location	Study years	Effect of women’s groups on neonatal mortality^1^	Number of liveborn infants included in analysis^2^	Number of pregnancies included in analysis^3^
Manandhar et al. 2004 [19]	Makwanpur, Nepal (rural)	2001–2003	OR 0.70 (95% CI 0.53, 0.94)	6,125	6,215
Tripathy et al. 2010 [10]	Saraikela Karshwan, West Singhbhum, and Keonjhar districts in Jharkhand and Odisha, India (rural)	2005–2008	OR 0.68 (95% CI 0.59, 0.78)	18,207	18,592
Azad et al. 2010 [11]	Bogra, Faridpur, and Moulavibazar districts, Bangladesh (rural)	2005–2007	RR 0.92 (95% CI 0.75, 1.12)	29,901^4^	30,628^4^
More et al. 2012 [17]	Mumbai, India (urban)	2006–2009	OR 1.48 (95% CI 1.06, 2.08)	15,075	15,071
Lewycka et al. 2013 [20]	Mchinji district, Malawi (rural)	2005–2009	OR 0.59 (95% CI 0.40, 0.86)^5^	9,497^5^	9,551^5^
Fottrell et al. 2013 [12]	Bogra, Faridpur, and Moulavibazar districts, Bangladesh (rural)	2009–2011	RR 0.62 (95% CI 0.43, 0.89)	17,308	17,640
Tripathy et al. 2016 [13]	Saraikela Karshwan, West Singhbhum, and Keonjhar districts in Jharkhand and Odisha, India (rural)	2009–2012	OR 0.69 (95% CI 0.53, 0.89)	7,042	7,100

^1^Published estimate comparing women’s group intervention to control group adjusting for covariates, unless otherwise specified.

^2^This number may differ from the number reported in the mortality estimate for the main trial paper as it includes liveborn infants with information collected as part of the survey questionnaire only.

^3^This number may differ from the number reported in the mortality estimate for the main trial paper as it includes pregnancies with information collected as part of the survey questionnaire only.

^4^Bangladesh 2005–2007 trial data used in this analysis include both women’s groups and traditional birth attendant training intervention and control areas.

^5^The Malawi trial was a 2-by-2 factorial cluster-randomised controlled trial of a women’s group intervention and an infant feeding programme. Results are from the women’s group intervention and control arms.

OR, odds ratio; RR, risk ratio.

We also included 2 studies that took place in the same geographical region of Bangladesh. The initial Bangladesh trial did not find evidence of a reduction in neonatal mortality for the women’s group intervention. This may have been due to very low coverage; only 3% of women reported attending women’s groups. The objective of the second trial was therefore to determine whether scaling up the coverage of women’s groups in the same geographical area would have an effect on neonatal mortality.

In all studies except the trials in Nepal and Malawi, the data collection systems involved a female, community-based key informant who reported births and deaths in her area, which covered a population ranging from 250 to 800 households. For the trials in Nepal and Malawi, the key informant identified women in pregnancy. This key informant met with a trained interviewer once a month. The interviewer verified the informant’s reports and paid her an incentive for each correct identification. In the Malawi trial, cluster enumerators, who were similar to key informants, were paid a monthly salary. Four to 6 weeks after delivery, the interviewer visited the home where a birth or death had been identified and collected information on the mother’s and family’s sociodemographic characteristics, as well as events in the antenatal, delivery, and postnatal periods using a structured questionnaire [9–12,17,19,20]. In the event of a maternal death, an interviewer or supervisor conducted a verbal autopsy with a relative or close friend [9,10,19].

### Measures

We selected outcomes representing a variety of important behavioural indicators in the antenatal, delivery, and postnatal periods, including the following: appropriate antenatal care, facility delivery, use of a safe delivery kit, hand washing by the birth attendant prior to delivery, use of a sterilised instrument to cut the umbilical cord, immediate wrapping of the newborn after delivery, delayed bathing of the newborn, immediate initiation of breastfeeding, and exclusive breastfeeding for the first 6 weeks after delivery. A safe delivery kit was normally available at low cost and typically included the following, at a minimum: soap, a clean string, a razor blade, and a plastic sheet [21]. Information collected in the different surveillance systems did not allow us to understand whether clean delivery practices were used independent of kit use. Although the Malawi trial collected data on clean delivery practices including hand washing by the birth attendant and use of a sterilised blade to cut the cord, the Ministry of Health’s position was to promote facility deliveries, and it was not acceptable for the study’s women’s groups to discuss clean home delivery practices or TBA training. Table 2 lists and defines the outcomes used in the analysis for each trial. We assessed the quality of evidence for each outcome using Grading of Recommendations Assessment, Development and Evaluation (GRADE) criteria, and these results can be found in S1 Table [22].

**Table 2:** Antenatal, delivery, and postnatal practices included in this analysis.

Health behaviour	Manandhar et al. 2004 [19]	Tripathy et al. 2010 [10]	Azad et al. 2010 [11]	More et al. 2012 [17]	Lewycka et al. 2013 [20]	Fottrell et al. 2013 [12]	Tripathy et al. 2016 [13]
**Healthcare seeking (all pregnant women)**							
At least 4 antenatal care visits with a skilled provider or at a health facility	Yes	Yes	Yes	Yes	Yes	Yes	Yes
Facility delivery (in the public or private sector)	Yes	Yes	Yes	Yes	Yes	Yes	Yes
**Clean delivery practices (for home deliveries only)**							
Birth attendant washes hands with soap prior to delivery	Yes	Yes	Yes	Yes	**No**	Yes	Yes
Birth attendant uses a safe delivery kit	Yes	Yes	Yes	**No**	**No**	Yes	Yes
Birth attendant cuts cord with new or sterile blade	Yes	Yes	Yes	Yes	**No**	Yes	**No**
**Thermal care (for home deliveries with live births)**							
Child is wrapped or put to skin within 10 minutes of delivery	**No**	Yes	Yes	**No**	Yes	Yes	Yes
Child is not bathed in first 24 hours after delivery	Yes	Yes	Yes	Yes	Yes	Yes	Yes
**Breastfeeding (all live births)**							
Child is breastfed within 1 hour of delivery	Yes	Yes	Yes	Yes	Yes	Yes	Yes
Exclusive breastfeeding for 6 weeks	Yes	Yes	Yes	Yes	Yes	Yes	Yes

‘Yes’ indicates information was collected for this outcome. ‘No’ indicates information was not collected for this outcome

The previous meta-analysis assessing the effect of women’s groups on mortality outcomes found that the coverage of groups and the proportion of pregnant women participating in them were key to mortality reduction [7]. As part of an additional analysis to test whether coverage also affected the success of the intervention in improving the behaviours of interest, we created a variable indicating whether a woman attended group meetings. Women who were allocated to the intervention arm and reported attending at least 1 group meeting were considered women’s group attendees.

### Statistical methods

We examined the prevalence of behaviours of interest either at baseline or, when this was not available, in the trial’s control arm. We also tabulated the prevalence of each behaviour by treatment arm and women’s group attendance (S2 Table).

We then used 2-stage meta-analysis techniques to estimate the effect of the women’s group intervention on behavioural outcomes. In the first stage, we used individual records to assess the effect of women’s groups on the selected outcomes separately for the different trials. We used logistic regression with random effects (*xtmelogit* command) in Stata to account for the clustered nature of the data [23]. For trials that used a stratified or paired trial design, we adjusted for the different strata/pairs using a dummy variable that we treated as a fixed effect. These analyses also adjusted for any baseline differences between the intervention and control arms that existed before the inception of any intervention activities (S1 Box). Although the Nepal trial collected information on whether a woman had a facility delivery, due to very few women having a facility delivery and the paired nature of this cluster-randomised trial, these models would not converge. Likewise, for the urban Indian trial, the model assessing the effect of groups on exclusive breastfeeding failed to converge because only 0.9% of women reported a positive response for this outcome. For the second stage of the meta-analysis, we used random effects models via the *metan* command in Stata [23]. We chose to do a 2-stage meta-analysis rather than use summary estimates from the published trials, as not all trials reported all behaviours of interest for our analysis, and this method also allowed us to adjust for additional confounders that were not accounted for in the original trial.

For trials with outcomes or covariates with greater than 10% missing data and significant differences in missingness between the control and intervention arms, we applied multiple imputation by chained equations (MICE) using the MI command in Stata, and assuming data were missing at random (MAR) [24]. Variables included in the MICE models were the outcome of interest, treatment arm, and covariates that were considered to be predictors of missingness [25,26]. We used a weighted sensitivity analysis using the selection model approach with multiple imputed data to test for modest departures from MAR [27–29]. In all instances, there was no evidence that missingness biased our main study findings.

### Women’s group attendance

For each of the studies, we used logistic regression with random effects (*xtmelogit* command) in Stata to assess associations between outcomes and the following categories of group attendance and allocation: women attending a group and allocated to the intervention arm, women not attending a group but allocated to the intervention arm, and women allocated to the control arm. Stata’s postestimation command ‘test’ was used to determine if there were significant differences in the ORs between (1) women who attended groups in the intervention arm versus women in the control arm and (2) women who did not attend groups in the intervention arm versus women in the control arm. Models were adjusted using methods similar to those described for the first stage of the meta-analysis in addition to including covariates likely to influence health behaviours and women’s group attendance: parity, maternal age, and maternal educational attainment (S1 Box). We identified these covariates by discussing the intervention with principal investigators and reviewing process evaluations and qualitative research on the women’s group interventions [14–16]. Although the second rural Indian trial (the Jharkhand Odisha Health Action Research [JOHAR] trial), the trial in urban India, and the Malawi trial adjusted for baseline differences, we did not adjust for baseline differences in this analysis as it would not have been possible for women to attend group meetings before their inception [13].

We chose not to do a pooled analysis of the associations between health behaviours and women’s group attendance because we expected both the determinants of women’s group attendance and the types of behaviours discussed at the women’s groups to differ substantially across trials, meaning that a single summary effect would not capture this heterogeneity adequately. All analyses were conducted in Stata 14 [23].

## Results

### General

The prevalence of antenatal, delivery, and postnatal health behaviours among women who were not exposed to the intervention (baseline period or control arm of the trial) differed substantially between studies (Table 3). For example, 2% of women delivered in health facilities in the control group of the trial in rural Nepal, compared with 84% of women in the baseline group in the urban India trial. Appropriate thermal care was uncommon in the first rural India trial, with only 12% of neonates being wrapped within 10 minutes of birth and only 17% having delayed bathing. Exclusive breastfeeding was rarely practised in urban India (1% at baseline, compared with between 20% and 94% at baseline or in the control arm in the other trials). Prevalence of behaviours for both the intervention and control arms can be found in S2 Table.

**Table 3:** Prevalence of selected health behaviours at baseline or in the control arms of women’s group trials.

Health behaviour of interest	Manandhar et al. 2004 [19] (rural Nepal)^1^	Tripathy et al. 2010 [10] (rural India)^2^	Azad et al. 2010 [11] (rural Bangladesh)^1^	More et al. 2012 [17] (urban India)^2^	Lewycka et al. 2013 [20] (rural Malawi)^2^	Fottrell et al. 2013 [12] (rural Bangladesh)^2^	Tripathy et al. 2016 [13] (rural India)^2^
**Healthcare seeking (all pregnant women)**							
At least 4 antenatal care visits with a skilled provider or at a health facility (%)	4.4	13.2	14.0	56.4	26.5	12.2	13.8
Delivered in a health care facility (%)	2.0	13.1	17.4	83.8	37.9	20.3	45.7
Number of pregnant women	3,266	4,655	15,099	5,208	2,560	12,996	3,277
**Clean delivery practices (home deliveries: all births)**							
Attendant washed hands (%)	54.4	29.3	77.0	69.3	80.3	81.3	58.7
Attendant used safe delivery kit (%)	4.0	10.0	16.5	—^3^	—^3^	12.5	4.1
Number of home deliveries for all pregnant women	3,199	3,947	12,349	842	1,558	6,221	1,775
**Postnatal care practices (home deliveries: all live births)**							
Attendant cut cord with new or sterile blade (%)	24.8	78.7	98.5	90.4	—^3^	99.0	—^3^
Baby was wrapped or kept warm within 10 minutes of delivery (%)	—^3^	12.3	19.3	—^3^	57.2	50.0	2.9
Baby was not bathed within 24 hours of delivery (%)	3.3	17.4	60.5	92.5	31.9	70.0	38.0
Number of home deliveries for all live births	3,162	3,840	12,134	839	1,542	10,136	1,710
**Breastfeeding (all live births)**							
Breastfed within 1 hour of birth (%)	53.3	27.9	61.7	45.8	73.7	62.0	81.5
Breastfed exclusively for 6 weeks following birth (%)	93.5	60.1	61.6	0.9	86.4	64.3	19.7
Number of live births	3,222	4,509	14,744	5,194	2,540	12,668	3,176

^1^Prevalence in control clusters.

^2^Prevalence in baseline data.

^3^Outcome not collected for this study.

### Effect of women’s groups on behavioural outcomes in the antenatal, delivery, and postnatal periods

**Fig 1:**
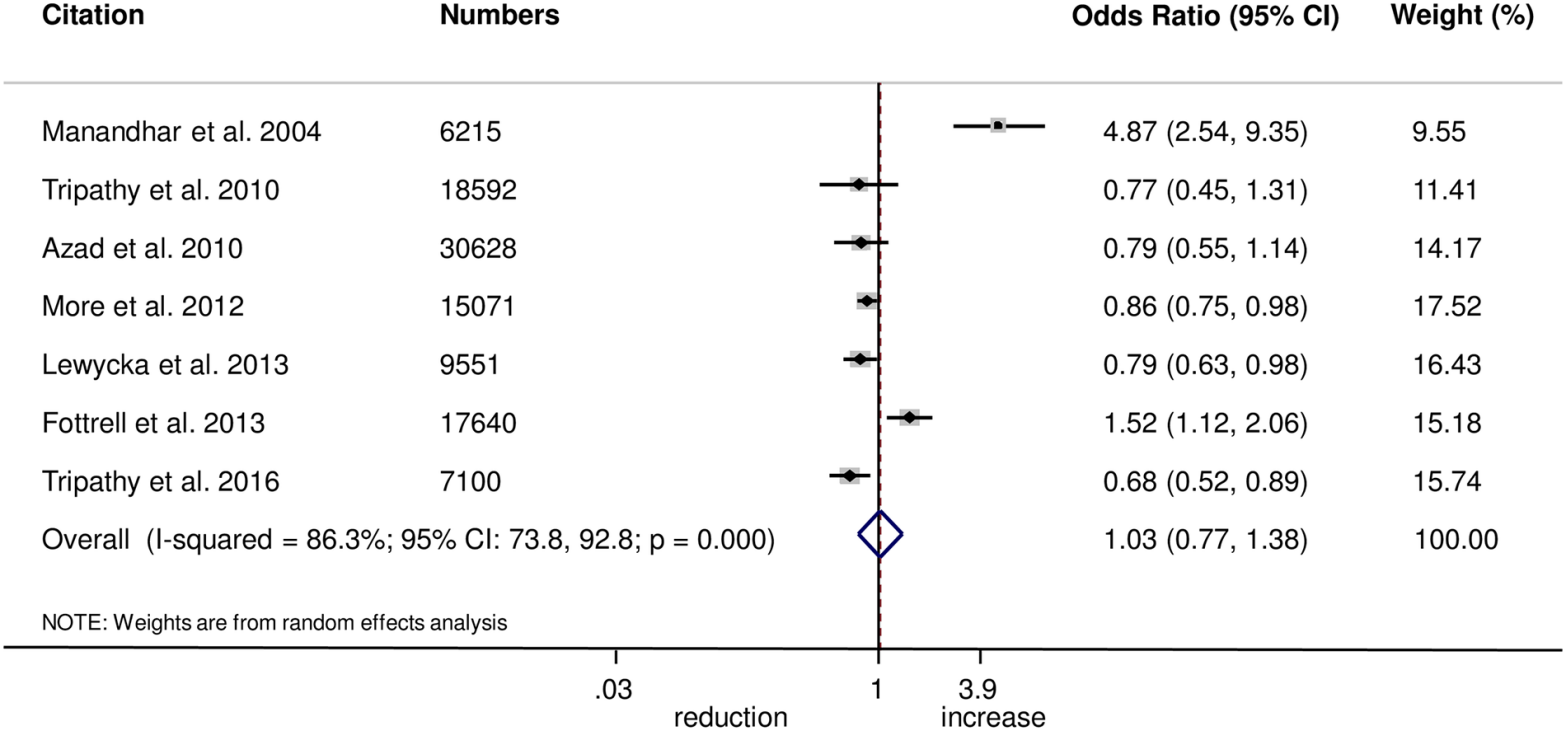
Meta-analysis of the effect of women’s groups on appropriate antenatal care.

**Fig 2:**
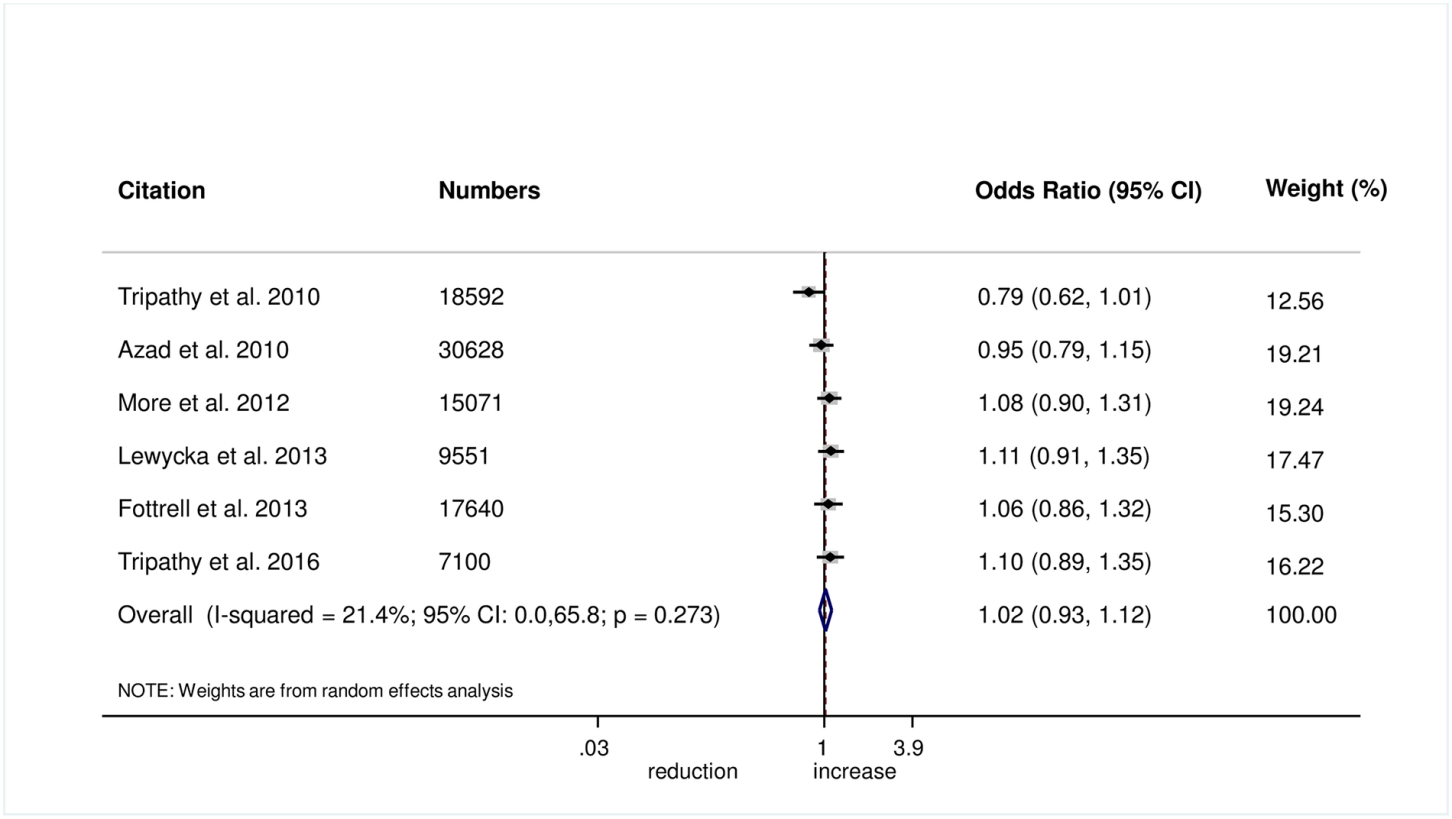
Meta-analysis of the effect of women’s groups on facility-based delivery.

The meta-analysis found no evidence that women’s groups improved the uptake of antenatal care (OR 1.03, 95% CI 0.77–1.38; *I*^2^ = 86.3%, 95% CI 73.8%–92.8%; Fig 1) (GRADE criteria: low; S1 Table) or health facility delivery (OR 1.02, 95% CI 0.93–1.12; *I*^2^ = 21.4%, 95% CI 0%–65.8%; Fig 2) (GRADE criteria: high; S1 Table), but we cannot rule out changes in the selectivity and speed of uptake of healthcare-seeking behaviours.

**Fig 3:**
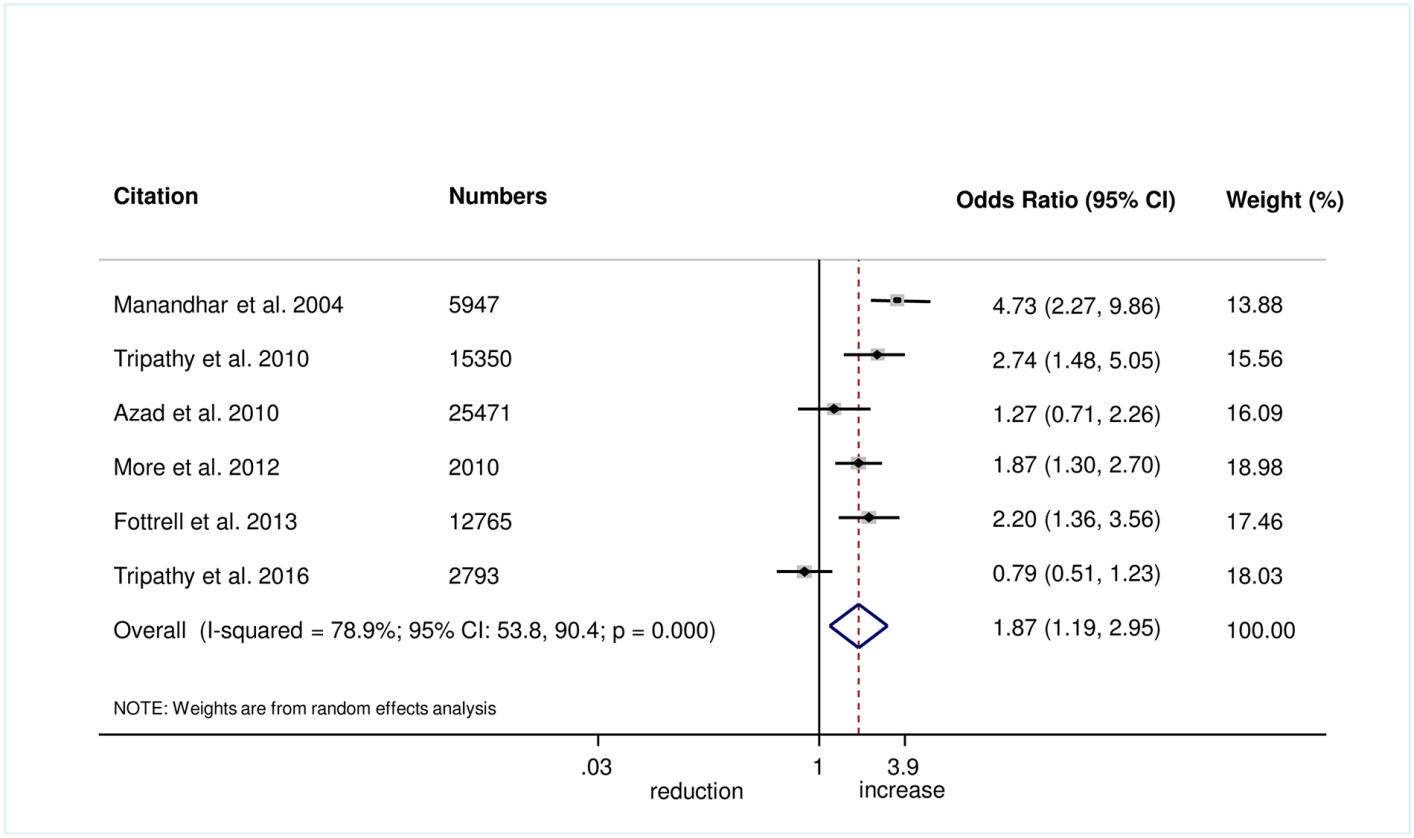
Meta-analysis of the effect of women’s groups on birth attendant washing hands prior to delivery for home deliveries.

**Fig 4:**
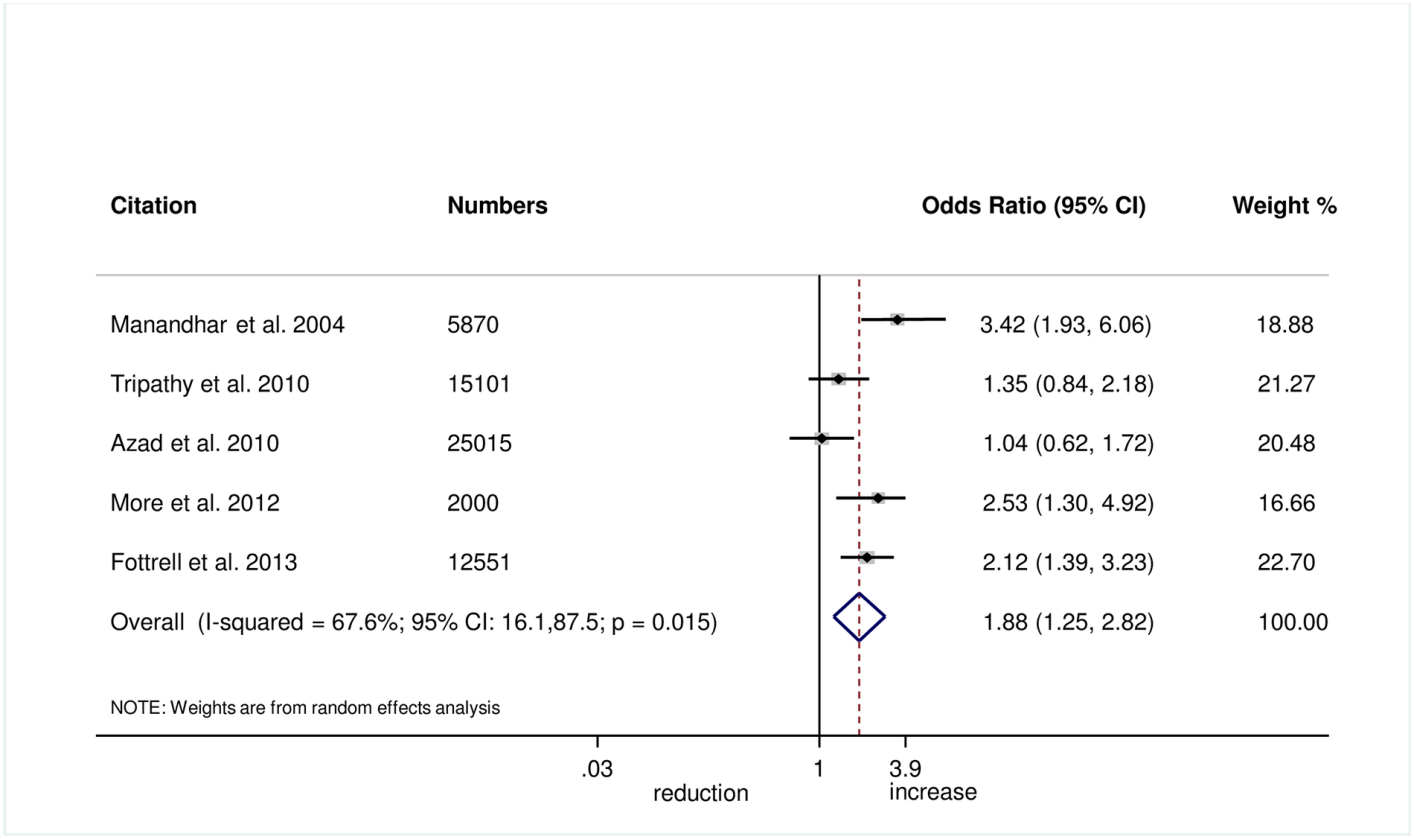
Meta-analysis of the effect of women’s groups on cutting the umbilical cord with a sterile instrument for home deliveries.

**Fig 5:**
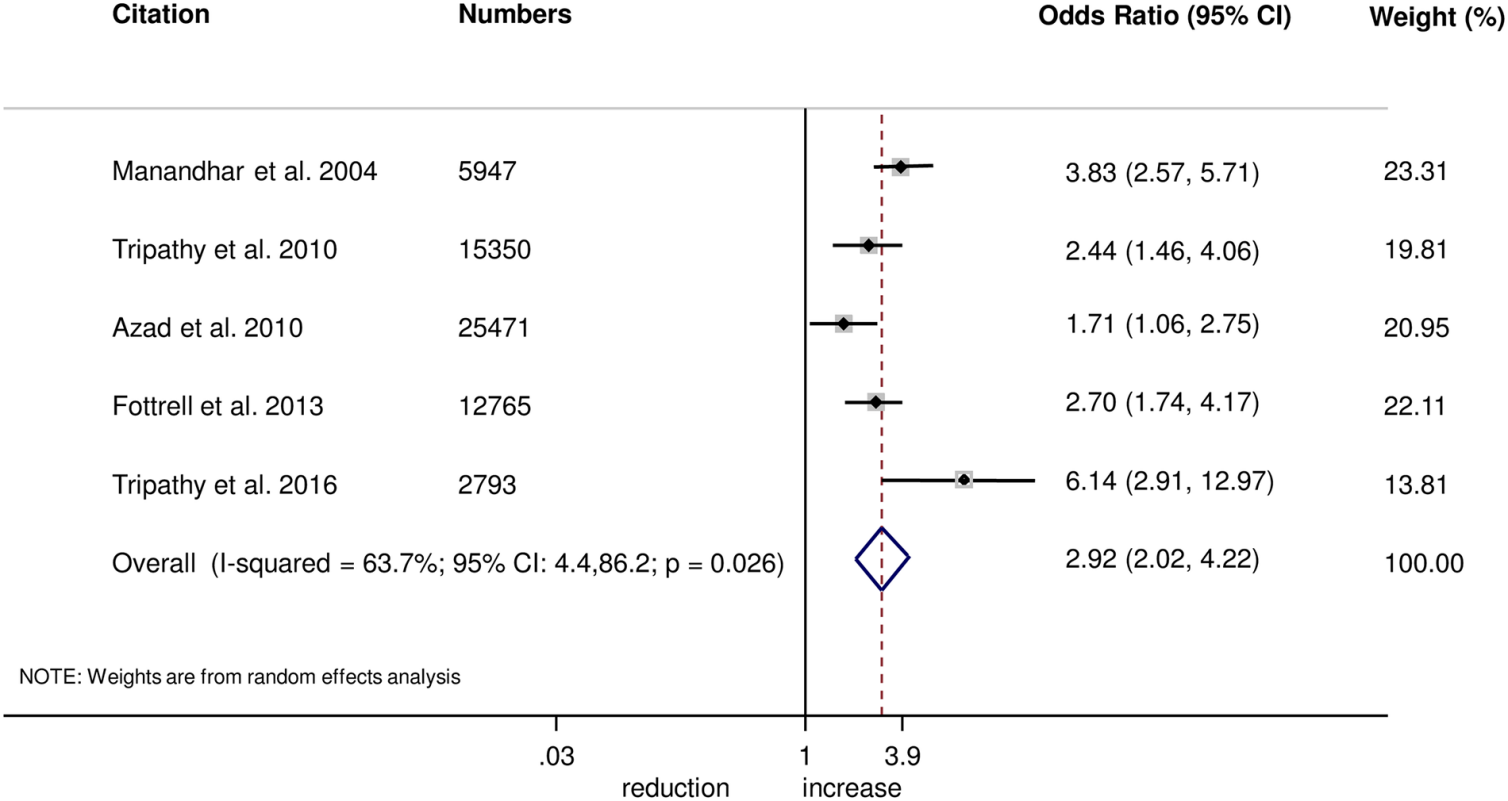
Meta-analysis of the effect of women’s groups on use of a safe delivery kit for home deliveries.

The meta-analysis suggests that women’s groups were effective in improving hygiene practices for home deliveries. Overall, there was evidence that women’s groups increased hand washing by birth attendants (OR 1.87, 95% CI 1.19–2.95; *I*^2^ = 78.9%, 95% CI 53.8%–90.4%; Fig 3) (GRADE criteria: low; S1 Table). There was also some evidence that women’s groups improved the use of new or sterile blades for cord cutting (OR 1.88, 95% CI 1.25–2.82; *I*^2^ = 67.6%, 95% CI 16.1%–87.5%; Fig 4) (GRADE criteria: low; S1 Table). There was moderate evidence that women’s groups improved the use of safe delivery kits (OR 2.92, 95% CI 2.02–4.22; *I*^2^ = 63.7%, 95% CI 4.4%–86.2%; Fig 5) (GRADE criteria: moderate; S1 Table).

**Fig 6:**
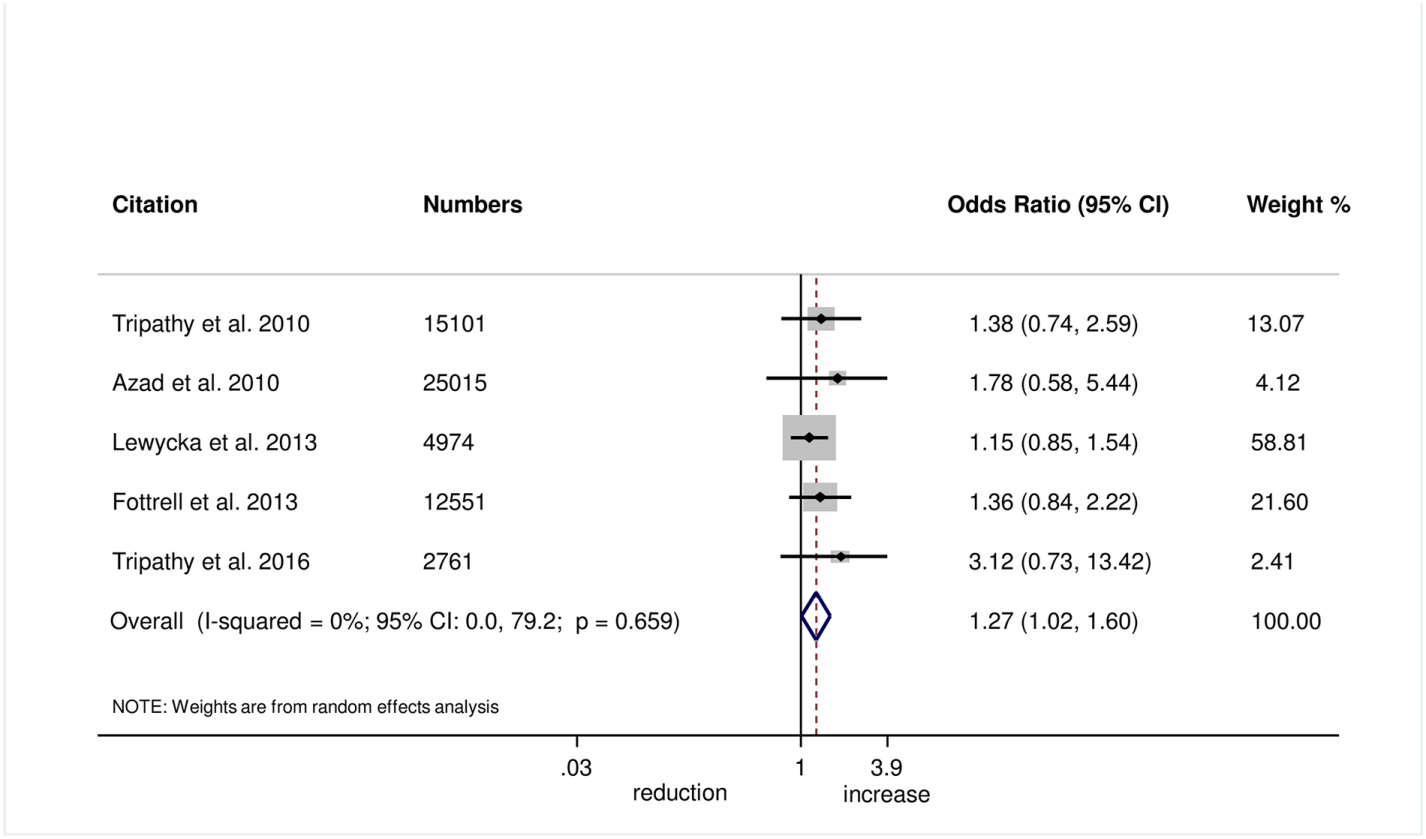
Meta-analysis of effect of women’s groups on wrapping the newborn within 10 minutes of delivery for home births.

**Fig 7:**
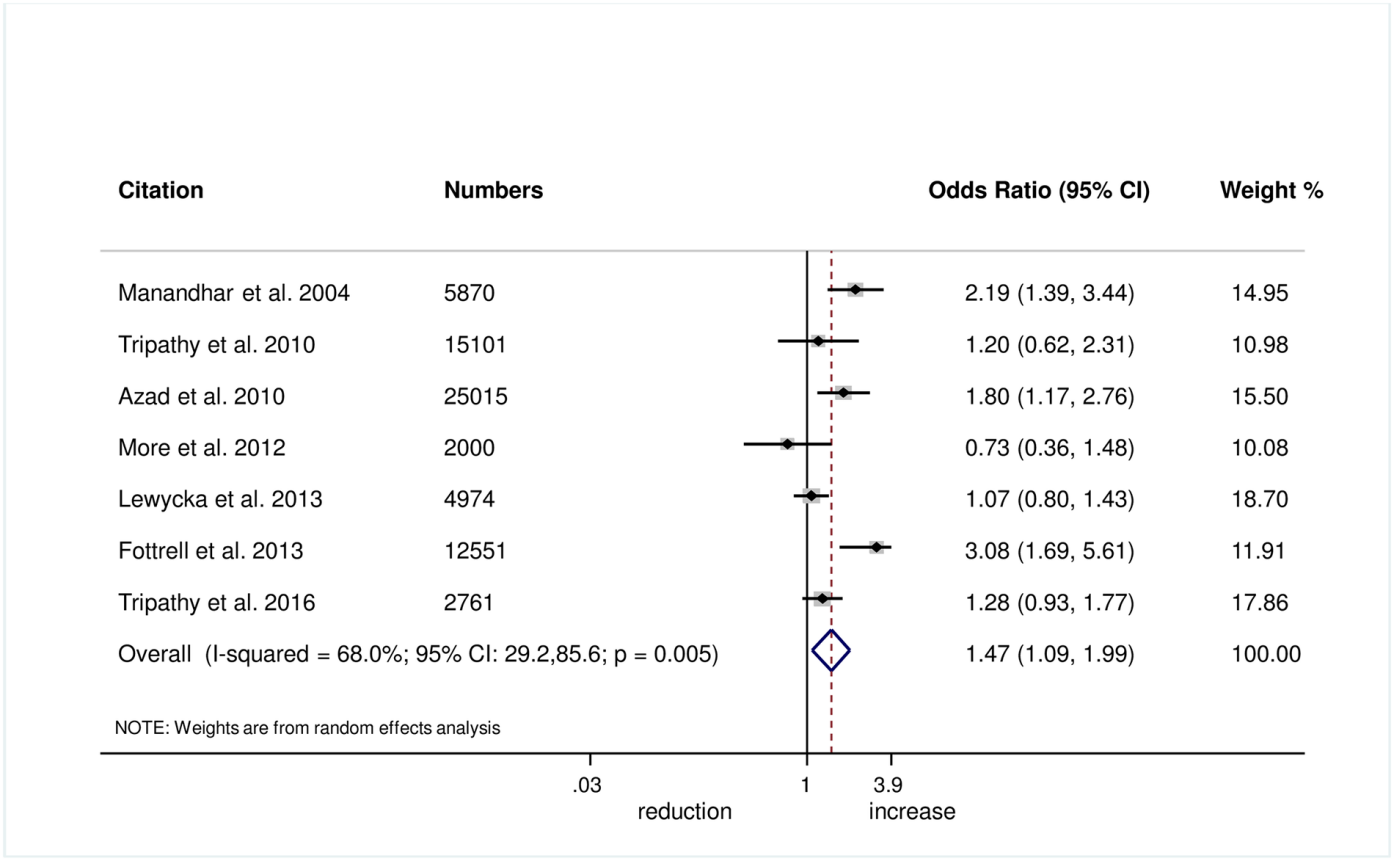
Meta-analysis of the effect of women’s groups on delaying bathing of a newborn for at least 24 hours after delivery for home deliveries.

Wrapping of the newborn within 10 minutes of birth was measured in 5 trials, and we found evidence of improvement in this practice with women’s groups (OR 1.27, 95% CI 1.02–1.60; *I*^2^ = 0.0%, 95% CI 0.0%–79.2%; Fig 6) (GRADE criteria: moderate; S1 Table). We also found some evidence of increases in delayed bathing (OR 1.47, 95% CI 1.09–1.90; *I*^2^ = 68%, 95% CI 29.2%–85.6%; Fig 7) (GRADE criteria: low; S1 Table).

**Fig 8:**
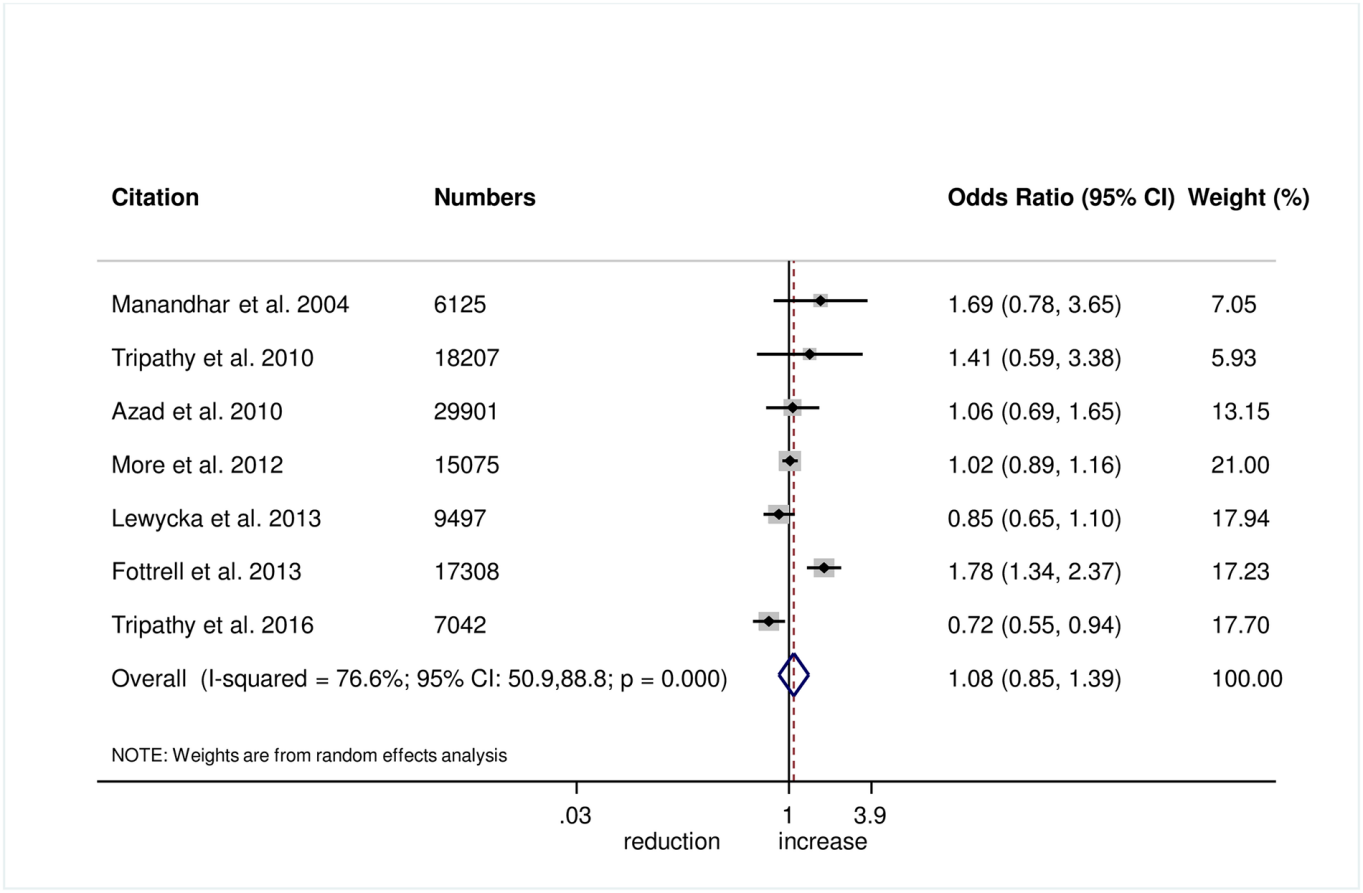
Meta-analysis of the effect of women’s groups on initiating breastfeeding within 1 hour of delivery.

**Fig 9:**
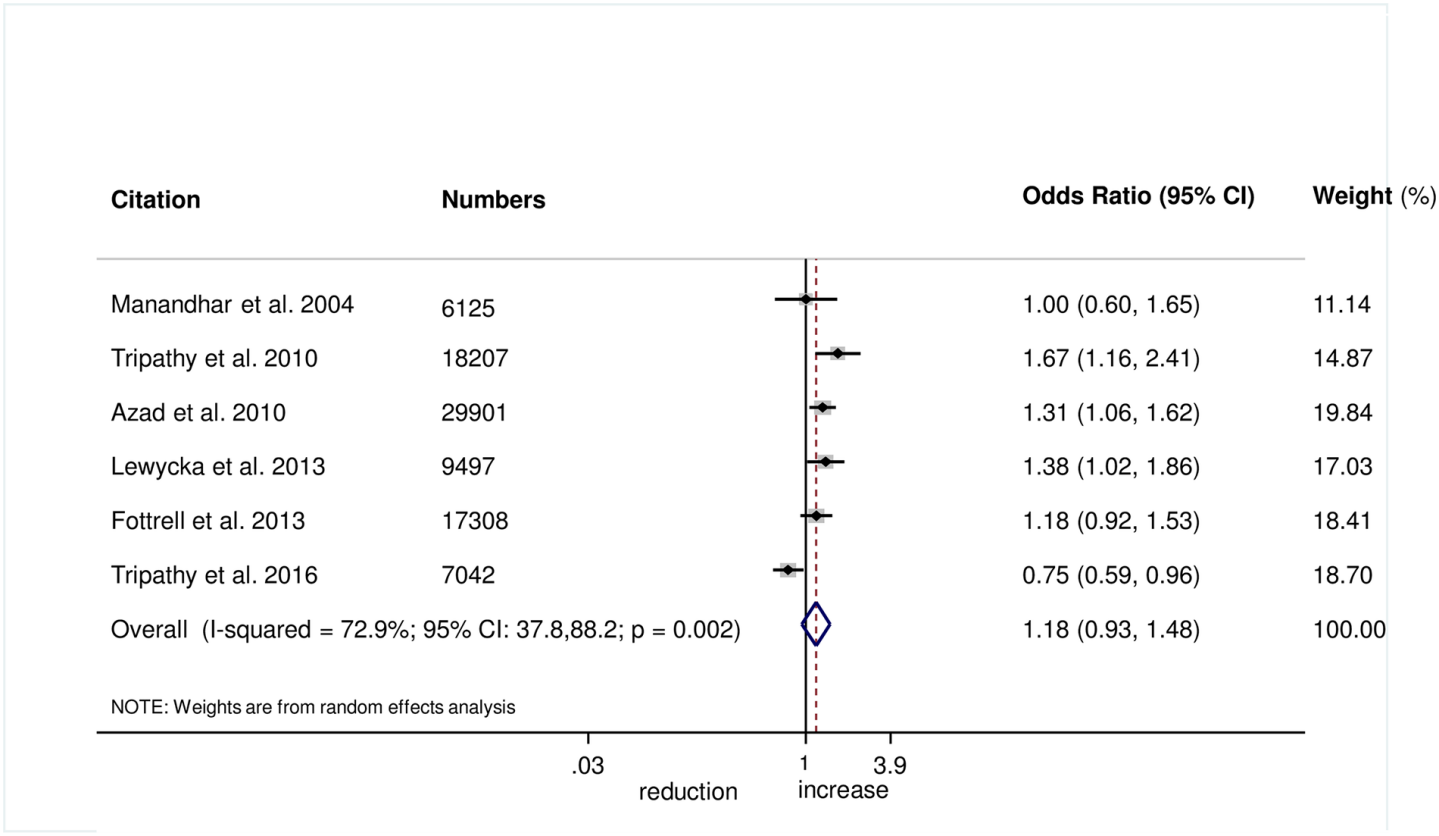
Meta-analysis of the effect of women’s groups on exclusive breastfeeding for 6 weeks following delivery.

There was no evidence that the intervention helped to improve breastfeeding within 1 hour of birth (OR 1.08, 95% CI 0.85–1.39; *I*^2^ = 76.6%, 95% CI 50.9%–88.8%; Fig 8) (GRADE criteria: low; S1 Table) or exclusive breastfeeding in the first 6 weeks of life (OR 1.18, 95% CI 0.93–1.48; *I*^2^ = 72.9%, 95% CI 37.8–88.2; Fig 9) (GRADE criteria: low; S1 Table).

### Effect of women’s group attendance on improving selected behaviours

We anticipated a positive relationship between exposure to the intervention and behaviour change, such that there would be a difference in the uptake of preventive and care-seeking behaviours between (1) women who attended groups in the intervention arm versus women in the control arm and (2) women who did not attend groups in the intervention arm versus women in the control arm. We expected that women who attended group meetings in the intervention arm would be more likely to modify their behaviours than women who were also in the intervention arm but did not attend group meetings. In most studies, and for the majority of behaviours, it was more likely that women who reported attending at least 1 group meeting were more likely to practise the behaviour in question. Detailed results can be found in Table 4.

**Table 4:** Differences in odds ratios (95% CIs) between (1) women who attended groups in the intervention arm versus women in the control arm and (2) women who did not attend groups in the intervention arm versus women in the control arm.

Health behaviour	Odds ratio (95% CI): intervention arm versus control arm						
	Manandhar et al. 2004 [19]	Tripathy et al. 2010 [10]	Azad et al. 2010 [11]	More et al. 2012 [17]	Lewycka et al. 2013 [20]	Fottrell et al. 2013 [12]	Tripathy et al. 2016 [13]
***Care-seeking behaviours***							
**Mother had at least 4 antenatal care visits with a skilled provider or at a health facility**							
Intervention arm non-attendees	4.67 (2.41, 9.03)	0.78 (0.45, 1.35)	**0.78 (0.55, 1.13)**	0.95 (0.79, 1.15)	**0.66 (0.35, 1.26)**	**1.31 (0.96, 1.80)**	0.66 (0.25, 1.75)
Intervention arm attendees	5.20 (2.66, 10.18)	0.72 (0.42, 1.25)	**1.72 (1.11, 2.66)**	1.18 (0.77, 1.81)	**0.79 (0.42, 1.50)**	**2.01 (1.46, 2.77)**	0.57 (0.22, 1.47)
**Delivered in institution/health facility**							
Intervention arm non-attendees	—^1^	**0.73 (0.56, 0.96)**	0.98 (0.81, 1.18)	0.87 (0.66, 1.23)	**0.99 (0.48, 2.03)**	**1.13 (0.91, 1.40)**	**0.89 (0.52, 1.52)**
Intervention arm attendees	—^1^	**0.86 (0.65, 1.14)**	1.04 (0.74, 1.46)	1.05 (0.53, 2.07)	**1.17 (0.57, 2.40)**	**0.99 (0.80, 1.24)**	**1.17 (0.70, 1.95)**
***Home care behaviours—clean delivery***							
**Birth attendant washed hands**							
Intervention arm non-attendees	**4.03 (1.90, 8.57)**	**2.51 (1.30, 4.85)**	**1.25 (0.70, 2.23)**	1.20 (0.81, 1.80)	—^2^	**1.90 (1.17, 3.10)**	0.77 (0.25, 2.39)
Intervention arm attendees	**6.11 (2.84, 13.15)**	**4.29 (2.22, 8.30)**	**2.59 (1.35, 4.99)**	0.81 (0.24, 2.75)	—^2^	**2.89 (1.75, 4.79)**	1.14 (0.38, 3.41)
**Safe delivery kit used**							
Intervention arm non-attendees	**2.65 (1.70, 4.12)**	**2.00 (1.17, 3.39)**	**1.71 (1.07, 2.74)**	—^3^	—^3^	**2.20 (1.40, 3.44)**	2.17 (0.87, 5.44)
Intervention arm attendees	**6.06 (3.90, 9.42)**	**3.70 (2.16, 6.30)**	**2.27 (1.35, 3.83)**	—^3^	—^3^	**2.72 (1.37, 5.85)**	1.04 (0.43, 2.43)
**Cord cut with sterile blade**							
Intervention arm non-attendees	**2.79 (1.56, 4.99)**	**1.22 (0.70, 2.12)**	1.03 (0.62, 1.72)	1.08 (0.60, 1.93)	—^3^	1.80 (1.12, 2.88)	—^3^
Intervention arm attendees	**4.69 (2.60 8.45)**	**2.47 (1.40, 4.35)**	1.89 (0.68, 5.25)	0.76 (0.09, 6.79)	—^3^	3.04 (1.50, 6.15)	—^3^
***Home care behaviours—thermal care***							
**Kept warm within 10 minutes of delivery**							
Intervention arm non-attendees	—^3^	1.47 (0.79, 2.75)	**1.76 (0.58, 5.36)**	—^3^	0.28 (0.07, 1.24)	**1.30 (0.79, 2.12)**	—^1^
Intervention arm attendees	—^3^	1.40 (0.75, 2.64)	**2.85 (0.91, 8.91)**	—^3^	0.32 (0.07, 1.41)	**1.49 (0.91, 2.45)**	—^1^
**Not bathed within 24 hours of birth**							
Intervention arm non-attendees	**1.53 (0.92, 2.56)**	**0.94 (0.48, 1.83)**	**1.79 (1.17, 2.74)**	—^4^	0.68 (0.19, 2.47)	**2.47 (1.33, 4.60)**	0.84 (0.34, 2.12)
Intervention arm attendees	**3.36 (2.02, 6.00)**	**1.98 (1.01, 3.85)**	**2.73 (1.68, 4.43)**	—^4^	0.71 (0.20, 2.56)	**4.78 (2.55, 8.95)**	1.24 (0.51, 3.02)
***Infant feeding***							
**Child was breastfed within 1 hour of delivery**							
Intervention arm non-attendees	1.61 (0.74, 3.49)	**1.22 (0.49, 3.05)**	**1.05 (0.68, 1.64)**	1.12 (1.02, 1.23)	2.15 (0.62, 4.45)	**1.68 (1.25, 2.26)**	1.26 (0.60, 2.65)
Intervention arm attendees	1.80 (0.83, 3.92)	**1.89 (0.76, 4.71)**	**1.34 (0.82, 2.18)**	1.18 (0.82, 1.69)	2.30 (0.66, 7.96)	**1.98 (1.46, 2.68)**	1.54 (0.75, 3.19)
**Child was exclusively breastfed for 6 weeks following birth**							
Intervention arm non-attendees	**0.89 (0.53, 1.47)**	**1.47 (1.06, 2.16)**	1.30 (1.05, 1.62)	—^5^	1.38 (0.33, 5.73)	**1.07 (0.83, 1.38)**	**0.48 (0.21, 1.07)**
Intervention arm attendees	**1.20 (0.70, 2.07)**	**2.29 (1.55, 3.39)**	1.48 (1.10, 1.99)	—^5^	1.22 (0.29, 5.06)	**1.43 (1.10, 1.86)**	**0.72 (0.32, 1.60)**

Attendees are women who were assigned to the intervention arm who attended at least 1 women’s group meeting; non-attendees are women who were assigned to the intervention arm but did not attend any women’s group meetings. Odds ratios are for these groups compared to women assigned to the control arm. Values in bold indicate behaviours that were affected by women’s group attendance or trial arm allocation (*p <* 0.05) and for which there was a difference between the odds ratios for attendees and non-attendees (*p <* 0.05 on Wald test comparing 2 parameters).

^1^Models would not converge.

^2^Outcome not discussed in women’s groups meetings.

^3^Outcome not measured for this trial.

^4^It was not possible to compute estimates due to the category for attended in the ‘allocated, attended’ variable having too few newborns that were not bathed early.

^5^There were too few breastfed children to estimate results.

Results suggested improvements for group attendees compared to non-attendees in increased antenatal care visits with a skilled provider in the first Bangladesh trial (OR comparing non-attendees to control: 0.78, 95% CI 0.55–1.13; OR comparing attendees to control: 1.72, 95% CI 1.11–2.66; *p*-value of adjusted Wald test comparing equality of parameters: *p <* 0.001) and the second Bangladesh trial (OR comparing non-attendees to control: 1.31, 95% CI 0.96–1.80; OR comparing attendees to control: 2.01, 95% CI 1.46–2.77; Wald test *p <* 0.001). Improvements for group attendees compared to non-attendees were also present in the rural Malawi trial (OR comparing non-attendees to control: 0.66, 95% CI 0.35–1.26; OR comparing attendees to control: 0.79, 95% CI 0.42–1.50; Wald test *p* = 0.019).

Facility delivery was more likely for group attendees compared to non-attendees for four trials. The first India trial demonstrated improved rates of facility delivery in group attendees compared to non-attendees (OR comparing non-attendees to control: 0.73, 95% CI 0.56–0.96; OR comparing attendees to control: 0.86, 95% CI 0.65–1.14; *p*-value of adjusted Wald test comparing equality of parameters: *p* = 0.027). The second Bangladesh trial also demonstrated a difference between attendees and non-attendees (OR comparing non-attendees to control: 1.13, 95% CI 0.91–1.40; OR comparing attendees to control: 0.99, 95% CI 0.80–1.24; Wald test *p* = 0.024). The JOHAR trial [13] in rural India also found a difference in facility-based deliveries when comparing group attendees and non-attendees (OR comparing non-attendees to control: 0.89, 95% CI 0.52–1.52; OR comparing attendees to control: 1.17, 95% CI 0.70–1.95; Wald test *p* = 0.017). Results from the trial in rural Malawi trial also suggest that facility deliveries were more likely for group attendees compared to non-attendees (OR comparing non-attendees to control: 0.99, 95% CI 0.48–2.03; OR comparing attendees to control: 1.17, 95% CI 0.57–2.40; Wald test *p* = 0.014).

Hand washing by the birth attendant prior to delivery was more likely for group attendees compared to non-attendees for all trials, except in the urban Indian trial and the JOHAR trial in rural India. Use of a safe delivery kit was more likely for group attendees compared to non-attendees in all trials except the JOHAR trial in rural India. Cutting the umbilical cord with a sterilised instrument was more likely for group attendees compared to non-attendees in all studies except the Bangladesh trials and the urban Indian trial.

Results suggested improvements for group attendees compared to non-attendees in wrapping the newborn within 10 minutes of delivery for the first Bangladesh trial (OR comparing non-attendees to control: 1.76, 95% CI 0.58–5.36; OR comparing attendees to control: 2.85, 95% CI 0.91–8.91; *p*-value of adjusted Wald test comparing equality of parameters: *p* < 0.001) and the second Bangladesh trial (OR comparing non-attendees to control: 1.30, 95% CI 0.79–2.12; OR comparing attendees to control: 1.49, 95% CI 0.91–2.45; Wald test *p* = 0.033). Not bathing a newborn within 24 hours of birth was more likely for group attendees compared to non-attendees for all trials except the Malawi trial and the JOHAR trial.

Breastfeeding a newborn within an hour of delivery was more likely for group attendees compared to non-attendees for the two rural Bangladesh trials and the first Indian trial. However, exclusively breastfeeding an infant for the first 6 weeks of life was more likely for group attendees in all trials except the first Bangladesh and the Malawi trial.

## Discussion

This meta-analysis suggests that women’s groups practising PLA improved home delivery and home care practices during birth and the postnatal period. We found evidence that women’s groups improved clean delivery practices for home deliveries, including the use of safe delivery kits, hand washing with soap by birth attendants prior to delivery, and clean cord cutting. We also found evidence that groups improved home care practices including wrapping newborn infants within 10 minutes of delivery and delaying the bathing of infants for at least 24 hours after delivery. There was no evidence that groups improved the uptake of facility deliveries, antenatal care, early breastfeeding, or exclusive breastfeeding for at least 6 weeks following delivery. Most of the estimates for the separate behaviours had a high degree of heterogeneity. The lack of consistency in improving behaviours across all trials was unsurprising given that groups were involved in a process where women identified, prioritised, and implemented solutions for problems that differed between settings and groups.

The previous meta-analysis that assessed the effect of groups on neonatal mortality suggested that the effect of the intervention was partly dependent on the proportion of pregnant women attending groups, and on the population coverage of the groups [7]. Our analysis tested whether the uptake of different behaviours was dependent on group attendance, and found improvements in some of the behaviours for women who attended groups compared to women who did not. Interestingly, although the first Bangladesh trial did not show any differences between the intervention and control arm in either neonatal mortality or the different care practices, results from our analysis demonstrated that attendees in the intervention arm were more likely to improve care practices compared to non-attendees in the intervention arm. This suggests that population coverage is an important factor in improving newborn health. Although not all outcomes measured suggested an improvement for group attendees compared to non-attendees, it is possible that some behaviours were not emphasised in the group meetings for some of the trials. It is also possible that some women did not attend meetings where particular behaviours were discussed. Finally, it is possible that we did not have an adequate sample size to test for these effects, given that the original trial papers were powered to detect a reduction in neonatal mortality and not a difference in behaviours, some of which would have had much higher intracluster correlation coefficients [13,30].

The main limitation of our analyses was the high degree of heterogeneity for most of the selected behaviours. This may be due to the limited number of trials involving women’s groups and the contextual heterogeneity of the settings in which they were conducted. Behaviours identified and promoted by groups as part of their solutions to improve maternal and newborn health were likely to be different in different settings, given that 5 of the trials took place in rural South Asia, 1 trial in urban India, and 1 trial in rural Malawi. The mechanisms that influenced improvements in neonatal and maternal health in these different settings are also likely to have been affected by local social and cultural norms and by environmentally specific conditions. For example, neonatal mortality rates are higher in winter in rural India, which may have resulted in more women’s groups identifying thermal care as an important practice, compared to groups in the Malawi trial [13,31].

Another potential limitation of this study was that most of the behaviours documented in the surveillance system were self-reported, and women in the intervention arm may have been more likely to report socially desirable behaviours compared to women in the control arm. This is a general limitation of self-reported data from trials that attempt to modify behaviours. Women in the intervention arm may also have been more likely to remember whether a care practice was used compared to women in the control arm. If women in the control arm were also less likely to practise the acceptable behaviour, this could have introduced bias. The sensitivity analysis testing the MAR assumption for the multiple imputation verified that our estimates were likely to be unbiased by missing data.

Our findings suggest that home care behaviours over which women and their families had greater control, including the use of clean delivery practices and appropriate thermal care, were more amenable to change than behaviours involving access to routine health services. Given findings from a previous study that found that clean delivery practices were associated with a reduction in neonatal mortality, it seems possible that the groups’ ability to improve clean delivery practices reduced cases of neonatal sepsis and that better thermal care practices reduced the danger of hypothermia, an important contributing factor to mortality [21]. The data on care seeking are less clear. Lack of improvement in most care-seeking practices may have been due to concerns around the availability, affordability, or quality of care in these areas [32–35].

We cannot rule out other mechanisms through which women’s groups may work, but these could not be examined in this study. For example, groups may change antenatal risk behaviours in diet, infection prevention, and substance use. Groups may also help families make more timely decisions about appropriate care seeking based on better information about the quality of care in local facilities. Finally, groups may also work by shifting a family’s ideas about complications from fatalism to response, and by improving access to resources and help in finding transport and care options [14–16].

Although our analysis identified improvements in some behaviours, there are still many unknowns. Attempting to understand the causal pathways behind the success or failure of complex interventions is important, and UK Medical Research Council guidance recommends a rigorous process evaluation to help gain insight into such mechanisms [36]. It is now possible to identify where more insight into the mechanisms behind the women’s groups success could be useful. For example, it may be useful to collect information on the number of group meetings attended by each individual participant, as this would provide better estimates of the dose response to exposure. In addition, recording the problems and strategies discussed at each meeting attended by individual women would provide a more sensitive measure of exposure.

Trials included in this meta-analysis took place between 2001 and 2012, which was a period of rapid change for maternal and neonatal health [37,38]. Not only did mortality decrease, there were also significant changes in behaviours on the pathway to mortality reduction. Importantly, there were substantial increases in facility deliveries and skilled birth attendance [1]. It is likely that different behaviours were emphasised at different time points between 2001 and 2012. For example, in 2005 the Indian government started the Janani Suraksha Yojana programme, a conditional cash transfer encouraging women to deliver in public health facilities. The Janani Suraksha Yojana has been responsible for increasing the proportion of deliveries occurring in facilities from 38% in 2005 to 74% in 2013 [39]. Likewise, in Malawi, facility deliveries increased nationally from 55% to 91% between 2000 and 2015 [40]. Results from the rural Indian trial taking place between 2005 and 2008 showed that groups did not have an impact on improving the proportion of women delivering in health facilities, but the JOHAR trial (2009–2012) found that groups improved the uptake of facility-based delivery. This may highlight one of the benefits of ‘agile’ interventions such as participatory women’s groups, which are dialogue-based rather than dependent on a fixed set of messages: they are flexible by design, which allows groups to respond to changes in the social environment and health system. The flexibility of women’s groups in offering context-specific solutions to problems suggests that this approach may also be appropriate for settings with a medium to high proportion of facility deliveries. For example, findings from a trial in Vietnam suggest that PLA using local stakeholder groups composed of health workers and other community workers may reduce neonatal mortality in areas with mainly facility-based deliveries and moderate levels of mortality [41].

A recent meta-analysis of community-based approaches to improve neonatal mortality found that community interventions had negligible effects in settings where mortality rates were less than 32 per 1,000 live births [42]. Findings from this meta-analysis also suggested that community interventions are less effective when facility-based deliveries are greater than 44% [42]. The authors further explained that in such contexts, unhealthy home care practices are easily addressable risk factors. These findings are supported by results of our-meta-analysis that showed improvements in crucial home care practices including clean deliveries and appropriate thermal care.

All trials included in this meta-analysis were conducted by University College London’s Institute for Global Health, with separate partner organisations responsible for leading the interventions and data collection. Lessons learned from the initial trials were used to improve subsequent studies. As an example, in the first Bangladesh trial, the population coverage of women’s groups was probably insufficient to achieve results. To address this, coverage was increased and a second trial conducted. Questions may arise as to the reproducibility of findings from the studies included in this meta-analysis, and whether PLA will be effective when brought to scale. These are valid concerns that are being addressed in scale-up initiatives, for example with accredited social health activists (ASHAs) and their supervisors supported by the National Health Mission in rural India. Results from the non-randomised, controlled evaluation of this initiative will help us better understand whether PLA will be effective when brought to scale.

The Global Strategy for Women’s, Children’s and Adolescents’ Health is a roadmap for ending preventable deaths (‘survive’), ensuring health and well-being (‘thrive’), and expanding enabling environments (‘transform’) [43]. The UN Secretary General has made ‘community empowerment’ the priority for the transformative component of this agenda [44]. Findings from our meta-analysis suggest that women’s groups practising PLA can improve care pathways that are key to reducing maternal and neonatal morbidity and mortality. Future research can help to assess whether such interventions can be used to address health-related issues along the continuum of care for women, children, and adolescents.

## Supporting information

S1 Box List of adjusted covariates used in different models. (DOCX)

S1 Table Results of GRADE scoring system used for chosen behavioural outcomes. (DOCX)

S2 Table Prevalence of behaviours among women allocated to the control arm, women allocated to the intervention arm and not attending women’s groups, and women allocated to the intervention arm and attending women’s groups. (DOCX)

S1 Dataset List of variables and associated value labels for dataset. (XLSX)

S2 Dataset Main dataset including all relevant variables. (XLSX)

## Abbreviations

GRADE: Grading of Recommendations Assessment, Development and Evaluation
JOHAR: Jharkhand Odisha Health Action Research
MAR: missing at random
OR: odds ratio
PLA: participatory learning and action
TBA: traditional birth attendant
