# Supplementary material for: Effects of women’s groups practising participatory learning and action on preventive and care-seeking behaviours to reduce neonatal mortality: A meta-analysis of cluster-randomised trials
Source: PLoS Med. 2017 Dec 5;14(12):e1002467. doi: 10.1371/journal.pmed.1002467 (PMC5716527; doi:10.1371/journal.pmed.1002467)
Supplement: S1 Box — (DOCX) [file pmed.1002467.s001.docx]

**Supporting Box 1. List of adjusted covariates included in different models estimating associations between women’s groups and behaviours in the antenatal, delivery, and postpartum period**

| **Citation** | **Location** | **Covariates adjusted in models estimating associations between women’s groups and behaviours in the antenatal, delivery, and postpartum period** | Covariates adjusted in models estimating associations between women’s group attendance and behaviours in antenatal, delivery, and postpartum periods |
| --- | --- | --- | --- |
| Manandhar et al. 2004 | Makwanpur, rural Nepal | Household assets, mother’s education, | Household assets, maternal education, parity, maternal age |
| Tripathy et al. 2010 | Jharkhand and Odisha, rural India | Mother’s religion, mother’s education, household assets, Hindu faith | Hindu faith, household assets, maternal education, parity, maternal age |
| Azad et al. 2010 | Bogra, Faridpur, Moulavibazar, rural Bangladesh | Mother’s age, mother’s education, household assets | Household assets, maternal education, parity, maternal age |
| Lewycka et al. 2013 | Mchinji, rural Malawi | Parity, household assets, baseline differences | Household assets, maternal education, parity, maternal age |
| More, et al. 2012 | Mumbai, urban India | Household assets, Muslim faith, baseline neonatal mortality rate | Household assets, maternal education, parity, maternal age, Muslim faith |
| Fottrell et al. 2013 | Bogra, Faridpur, Moulavibazar, rural Bangladesh | Mother’s age, mother’s education, household assets | Household assets, maternal education, parity, maternal age |
| Tripathy et al. 2016 | Jharkhand and Odisha, rural India | Baseline differences | Household assets, maternal education, parity, maternal age |

Description of covariate:

1. Household assets in Nepal (categorical variable: 0=none; 1=clock, radio, iron, bicycle; 2=hand tractor, sewing machine, camera; 3=bus, truck, motorcycle, television)

2. Maternal education for all trials (categorical variable: 1=no education, 2=primary education, 3=secondary education and above)

3. Parity for all trials (categorical variable: 1=primiparous, 2=multiparous, 3=grand multiparous)

4. Maternal age for all trials (discrete variable)

5. Hindu faith for first India trial (dichotomous variable: 0=not Hindu/1=Hindu)

6. Household assets for all trials except Nepal and Malawi (categorical variable reported in quintiles)

7. Household assets in Malawi (categorical variable: 0=no assets, 1=one asset, 2=two assets or more)

8. Muslim faith for Mumbai trial only (0=not Muslim, 1=Muslim)
