# Supplementary material for: Effects of women’s groups practising participatory learning and action on preventive and care-seeking behaviours to reduce neonatal mortality: A meta-analysis of cluster-randomised trials
Source: PLoS Med. 2017 Dec 5;14(12):e1002467. doi: 10.1371/journal.pmed.1002467 (PMC5716527; doi:10.1371/journal.pmed.1002467)
Supplement: S1 Table — (DOCX) [file pmed.1002467.s002.docx]

**Supporting Table 1. Results of GRADE scoring system used for chosen behavioural outcomes**

| **Women's groups intervention using PLA to improve the following behavioural outcomes** | **Number of studies** | **Study design** | **Quality** | **Consistency^2^** | **Generalisability** | **Effect size –  Odds ratio (95% CI)** | **GRADE of evidence ^1^** |
| --- | --- | --- | --- | --- | --- | --- | --- |
| Antenatal care | 7 | Randomised | Problem with  one element ^1^ | lack of agreement | generalisable | 1.03, 0.77-1.38 | Low |
| Facility deliveries | 6 | Randomised | No problems | almost all studies similar | generalisable | 1.02, 0.93-1.12 | High |
| Hand washing with soap | 6 | Randomised | Problem with  one element ^1^ | lack of agreement | generalisable | 1.87, 1.19-2.95 | Low |
| Sterile blade to cut the umbilical cord | 5 | Randomised | Problem with  one element ^1^ | lack of agreement | generalisable | 1.88, 1.25-2.82 | Low |
| Safe delivery kit use | 5 | Randomised | Problem with  one element ^1^ | lack of agreement | generalisable | 2.92, 2.02-4.2 | Moderate |
| Wrapping of newborn within 10 minutes | 5 | Randomised | Problem with  one element ^1^ | almost all studies similar | generalisable | 1.27, 1.02-1.60 | Moderate |
| Delayed bathing of newborn | 7 | Randomised | Problem with  one element ^1^ | lack of agreement | generalisable | 1.47, 1.09-1.90 | Low |
| Breastfeeding of newborn within one hour of delivery | 7 | Randomised | Problem with  one element ^1^ | lack of agreement | generalisable | 1.08, 0.85-1.39 | Low |
| Exclusive breastfeeding for 6 weeks | 6 | Randomised | Problem with  one element ^1^ | lack of agreement | generalisable | 1.18, 0.93-1.48 | Low |

^1^ Recall bias

^2^ Consistency based on I^2^ statistic: Lack of agreement (I2>60% ); almost all studies are similar (I2=0% - 21%)
