# Supplementary material for: Effects of women’s groups practising participatory learning and action on preventive and care-seeking behaviours to reduce neonatal mortality: A meta-analysis of cluster-randomised trials
Source: PLoS Med. 2017 Dec 5;14(12):e1002467. doi: 10.1371/journal.pmed.1002467 (PMC5716527; doi:10.1371/journal.pmed.1002467)
Supplement: S2 Table — (DOCX) [file pmed.1002467.s003.docx]

## Supporting Table 2: Prevalence of behaviours among women in control clusters, women allocated to women's group clusters, and women attending women's groups

| **Behavioural outcomes of interest** | **Manandhar et al. 2004  (rural Nepal)** | | | | | **Tripathy et al. 2010  (rural India)** | | | | | **Azad et al. 2010  (rural Bangladesh)** | | | | | **More et al. 2012  (urban India)** | | | | |
| --- | --- | --- | --- | --- | --- | --- | --- | --- | --- | --- | --- | --- | --- | --- | --- | --- | --- | --- | --- | --- |
|  | All women | | Intervention | | Total | All women | | Intervention | | Total | All women | | Intervention | | Total | All women | | Intervention | | Total |
|  | Cont- rol | Interv-ention | Did  not attend | Atten- ded |  | Cont- rol | Interv-ention | Did  not attend | AttHi Everyone,  Sorry for the delay en ded |  | Cont- rol | Interv-ention | Did  not attend | Atten- ded |  | Cont- rol | Interv-ention | Did  not attend | Atten- ded |  |
| **Health care seeking behaviours for all pregnancies** |  |  |  |  |  |  |  |  |  |  |  |  |  |  |  |  |  |  |  |  |
| At least four ANC visits with a skilled provider or at a health facility |  |  |  |  |  |  |  |  |  |  |  |  |  |  |  |  |  |  |  |  |
| No | 95.6 | 86.6 | 86.2 | 87.4 | 91.3 | 76.9 | 84.9 | 83.3 | 87.7 | 81.1 | 86 | 88.6 | 88.7 | 82.5 | 87.3 | 33.3 | 36.1 | 36.1 | 30.8 | 34.7 |
| Yes | 4.4 | 13.4 | 13.8 | 12.6 | 8.7 | 23.1 | 15.1 | 16.7 | 12.3 | 18.9 | 14 | 11.4 | 11.3 | 17.5 | 12.7 | 66.7 | 63.9 | 63.9 | 69.2 | 65.3 |
| Delivery in institution/health facility |  |  |  |  |  |  |  |  |  |  |  |  |  |  |  |  |  |  |  |  |
| Not delivered in health facility | 98 | 93.2 | 93.3 | 93.1 | 95.7 | 79.6 | 85.6 | 84.5 | 87.3 | 82.7 | 82.6 | 85.4 | 85.3 | 87.7 | 84 | 12.8 | 13.8 | 13.9 | 10 | 13.3 |
| Delivered in health facility | 2 | 6.8 | 6.7 | 6.9 | 4.3 | 20.4 | 14.4 | 15.5 | 12.7 | 17.3 | 17.4 | 14.6 | 14.7 | 12.3 | 16 | 87.2 | 86.2 | 86.1 | 90 | 86.7 |
| Total (all women) | 3,267 | 2,948 | 1,883 | 1,065 | 6,215 | 8,998 | 9,594 | 6,043 | 3,551 | 18,592 | 15,099 | 15,529 | 15,051 | 475 | 30,625 | 7,472 | 7,599 | 7,469 | 130 | 15,071 |
| **Home delivery practices for all pregnancies, prior to birth of newborn** |  |  |  |  |  |  |  |  |  |  |  |  |  |  |  |  |  |  |  |  |
| Attendant washed hands |  |  |  |  |  |  |  |  |  |  |  |  |  |  |  |  |  |  |  |  |
| No | 45.6 | 19 | 19.5 | 18.1 | 31.2 | 76.2 | 57.3 | 59.6 | 53.7 | 66.1 | 23 | 21.2 | 21.4 | 14 | 22.1 | 38.6 | 33.6 | 33.5 | 46.2 | 723 |
| Yes | 54.4 | 81 | 80.5 | 81.9 | 68.8 | 23.8 | 42.7 | 40.4 | 46.3 | 33.9 | 77 | 78.8 | 78.6 | 86 | 77.9 | 61.4 | 66.4 | 66.5 | 53.8 | 1286 |
| Attendant used safe/clean delivery kit |  |  |  |  |  |  |  |  |  |  |  |  |  |  |  |  |  |  |  |  |
| No | 96 | 83.4 | 87.2 | 76.7 | 90.2 | 81.7 | 67.7 | 70 | 63.9 | 74.2 | 83.5 | 74.6 | 74.7 | 72.4 | 78.9 | ^1^ | ^1^ | ^1^ | ^1^ | ^1^ |
| Yes | 4 | 16.6 | 12.8 | 23.3 | 9.8 | 18.3 | 32.3 | 30 | 36.1 | 25.8 | 16.5 | 25.4 | 25.3 | 27.6 | 21.1 | ^1^ | ^1^ | ^1^ | ^1^ | ^1^ |
| Total (home deliveries for all women) | 3200 | 2747 | 1756 | 991 | 5947 | 7152 | 8198 | 5103 | 3095 | 15350 | 12349 | 13122 | 12707 | 413 | 25469 | 960 | 1050 | 1037 | 13 | 2010 |

| **Behavioural outcomes of interest** | **Manandhar et al. 2004  (rural Nepal)** | | | | | **Tripathy et al. 2010  (rural India)** | | | | | **Azad et al. 2010  (rural Bangladesh)** | | | | | **More et al. 2012  (urban India)** | | | | |
| --- | --- | --- | --- | --- | --- | --- | --- | --- | --- | --- | --- | --- | --- | --- | --- | --- | --- | --- | --- | --- |
|  | All women | | Intervention | | Total | All women | | Intervention | | Total | All women | | Intervention | | Total | All women | | Intervention | | Total |
|  | Cont- rol | Interv-ention | Did  not attend | Atten- ded |  | Cont- rol | Interv-ention | Did  not attend | Atten ded |  | Cont- rol | Interv-ention | Did  not attend | Atten- ded |  | Cont- rol | Interv-ention | Did  not attend | Atten- ded |  |
| **Postnatal home delivery practices for all liveborn newborns** |  |  |  |  |  |  |  |  |  |  |  |  |  |  |  |  |  |  |  |  |
| Attendant cut cord with new or sterile blade |  |  |  |  |  |  |  |  |  |  |  |  |  |  |  |  |  |  |  |  |
| No | 75.2 | 45.1 | 46.3 | 43 | 61.4 | 15.7 | 12.6 | 13.7 | 10.8 | 14 | 1.5 | 1.9 | 1.9 | 1.3 | 1.7 | 7.3 | 8.1 | 8.1 | 8.3 | 7.7 |
| Yes | 24.8 | 54.9 | 53.7 | 57 | 38.6 | 84.3 | 87.4 | 86.3 | 89.2 | 86 | 98.5 | 98.1 | 98.1 | 98.7 | 98.3 | 92.7 | 91.9 | 91.9 | 91.7 | 92.3 |
| Baby was kept warm or wrapped within 10 minutes of delivery |  |  |  |  |  |  |  |  |  |  |  |  |  |  |  |  |  |  |  |  |
| No | ^1^ | ^1^ | ^1^ | ^1^ | ^1^ | 91 | 90.6 | 90.2 | 91.2 | 90.7 | 80.7 | 70.5 | 70.7 | 63,6 | 75.5 | ^1^ | ^1^ | ^1^ | ^1^ | ^1^ |
| Yes | ^1^ | ^1^ | ^1^ | ^1^ | ^1^ | 9 | 9.4 | 9.8 | 8.8 | 9.3 | 19.3 | 29.5 | 29.3 | 36.4 | 24.5 | ^1^ | ^1^ | ^1^ | ^1^ | ^1^ |
| Baby was bathed within 24 hours of delivery |  |  |  |  |  |  |  |  |  |  |  |  |  |  |  |  |  |  |  |  |
| No | 96.7 | 91.5 | 92.8 | 89 | 94.3 | 77.4 | 73 | 75.7 | 68.5 | 75 | 39.5 | 28.8 | 29 | 22.8 | 34 | 4.1 | 5.7 | 5.8 | 0 | 5 |
| Yes | 3.3 | 8.5 | 7.2 | 11 | 5.7 | 22.6 | 27 | 24.3 | 31.5 | 25 | 60.5 | 71.2 | 71 | 77.3 | 66 | 95.9 | 94.3 | 94.2 | 100 | 95 |
| Total (home deliveries for all livebirths) | 3,162 | 2,708 | 1,733 | 975 | 5,870 | 7,048 | 8,053 | 5,011 | 3,042 | 15,101 | 12,134 | 12,881 | 12,447 | 402 | 25,013 | 952 | 1,048 | 1,036 | 12 | 2,000 |
| **Breastfeeding practices for all liveborn newborns** |  |  |  |  |  |  |  |  |  |  |  |  |  |  |  |  |  |  |  |  |
| Breastfed within 1 hour of delivery |  |  |  |  |  |  |  |  |  |  |  |  |  |  |  |  |  |  |  |  |
| Not breastfed within 1 hour | 46.7 | 38.6 | 38.8 | 38.1 | 42.9 | 78.6 | 75.2 | 75.3 | 75 | 76.8 | 38.3 | 37.5 | 37.7 | 32.2 | 37.9 | 51.8 | 47.9 | 47.9 | 48.4 | 49.8 |
| Breastfed within 1 hour | 53.3 | 61.4 | 61.2 | 61.9 | 57.1 | 21.4 | 24.8 | 24.7 | 25 | 23.2 | 61.7 | 62.5 | 62.3 | 67.8 | 62.1 | 48.2 | 52.1 | 52.1 | 51.6 | 50.2 |
| Exclusive breastfeeding for 6 weeks following birth |  |  |  |  |  |  |  |  |  |  |  |  |  |  |  |  |  |  |  |  |
| Not breastfed exclusively for 6w | 6.5 | 8.7 | 10 | 6.4 | 7.5 | 31.4 | 21.1 | 23.8 | 16.4 | 26.1 | 38.4 | 31.7 | 31.8 | 28.8 | 35 | ^1^ | ^1^ | ^1^ | ^1^ | ^1^ |
| Breastfed exclusively for 6w | 94.5 | 91.3 | 90 | 93.6 | 92.5 | 68.6 | 78.9 | 76.2 | 83.6 | 73.9 | 61.6 | 68.3 | 68.2 | 71.2 | 65 | ^1^ | ^1^ | ^1^ | ^1^ | ^1^ |
| Total (live births) | 3,222 | 2,902 | 1,856 | 1,046 | 6,124 | 8,819 | 9,388 | 5,912 | 3,476 | 18,207 | 14,744 | 15,157 | 14,699 | 455 | 29,898 | 7,478 | 7,597 | 7,466 | 131 | 15,075 |

## Supplementary Table 2 (continued): Prevalence of behaviours among women in control clusters, women allocated to women's group clusters, and women attending women's groups

| **Behavioural outcomes** | **Lewycka et al. 2013  (rural Malawi)** | | | | | **Fottrell et al. 2015  (rural Bangladesh)** | | | | | **Tripathy et al. 2016** | | | | |
| --- | --- | --- | --- | --- | --- | --- | --- | --- | --- | --- | --- | --- | --- | --- | --- |
|  | All women | | Intervention | | Total | All women | | Intervention | | Total | All women | | Intervention | | Total |
|  | Control | Interv-ention | Did  not attend | Atten- ded |  | Cont- rol | Interv-ention | Did  not attend | Atten- ded |  | Cont- rol | Interv-ention | Did  not attend | Atten-ded |  |
| **Health care seeking behaviours for all pregnancies** |  |  |  |  |  |  |  |  |  |  |  |  |  |  |  |
| At least four ANC visits with a skilled provider or at a health facility |  |  |  |  |  |  |  |  |  |  |  |  |  |  |  |
| No | 69.3 | 76.2 | 77.1 | 76 | 72.5 | 85.3 | 81.4 | 82.1 | 80.2 | 83.3 | 80 | 80.6 | 84.5 | 78.6 | 80.3 |
| Yes | 30.7 | 23.8 | 22.9 | 24 | 27.5 | 14.7 | 18.6 | 17.9 | 19.8 | 16.7 | 20 | 19.4 | 15.5 | 21.4 | 19.7 |
| Delivery in institution/health facility |  |  |  |  |  |  |  |  |  |  |  |  |  |  |  |
| Not delivered in health facility | 56.5 | 49.6 | 50 | 49.8 | 53.7 | 71.7 | 73 | 71.3 | 76 | 72.4 | 43.9 | 35 | 43 | 30.9 | 39.3 |
| Delivered in health facility | 43.5 | 50.4 | 50 | 50.2 | 46.3 | 28.3 | 27 | 28.7 | 24 | 27.6 | 56.1 | 65 | 57 | 69.1 | 60.7 |
|  |  |  |  |  |  |  |  |  |  |  |  |  |  |  |  |
| Total (all women) | 4,943 | 4,608 | 1,789 | 1,856 | 8,588 | 8,678 | 8,962 | 5,663 | 3,299 | 17,640 | 3,464 | 3,636 | 1,215 | 2,421 | 7,100 |
| **Home delivery practices for all pregnancies, prior to birth of newborn** |  |  |  |  |  |  |  |  |  |  |  |  |  |  |  |
| Attendant washed hands |  |  |  |  |  |  |  |  |  |  |  |  |  |  |  |
| No | ^2^ | ^2^ | ^2^ | ^2^ | ^2^ | 16 | 8.4 | 9.6 | 6.7 | 12 | 48.4 | 50 | 62.7 | 41.3 | 49.1 |
| Yes | ^2^ | ^2^ | ^2^ | ^2^ | ^2^ | 84 | 91.6 | 90.4 | 93.3 | 88 | 51.6 | 50 | 37.3 | 58.7 | 50.9 |
| Attendant used safe/clean delivery kit |  |  |  |  |  |  |  |  |  |  |  |  |  |  |  |
| No | ^1^ | ^1^ | ^1^ | ^1^ | ^1^ | 85 | 71.5 | 72.7 | 69.5 | 78 | 98 | 96.5 | 96 | 96.8 | 97.3 |
| Yes | ^1^ | ^1^ | ^1^ | ^1^ | ^1^ | 15 | 28.5 | 27.3 | 30.5 | 22 | 2 | 2.5 | 4 | 3.2 | 2.7 |
|  |  |  |  |  |  |  |  |  |  |  |  |  |  |  |  |
| Total (home deliveries for all women) | 2,740 | 2,258 | 893 | 922 | 4,555 | 6,221 | 6,554 | 4,037 | 2,507 | 12,765 | 1,521 | 1,272 | 523 | 749 | 2,793 |

## Supplementary Table 2 (continued): Prevalence of behaviours among women in control clusters, women allocated to women's group clusters, and women attending women's groups

| **Behavioural outcomes** | **Lewycka et al. 2013  (rural Malawi)** | | | | | **Fottrell et al. 2015  (rural Bangladesh)** | | | | | **Tripathy et al. 2016** | | | | |
| --- | --- | --- | --- | --- | --- | --- | --- | --- | --- | --- | --- | --- | --- | --- | --- |
|  | All women | | Intervention | | Total | All women | | Intervention | | Total | All women | | Intervention | | Total |
|  | Cont- rol | Interv-ention | Did  not attend | Atten- ded |  | Cont- rol | Interv-ention | Did  not attend | Atten- ded |  | Cont- rol | Interv-ention | Did  not attend | Atten-ded |  |
| **Postnatal home delivery practices for all liveborn newborns** |  |  |  |  |  |  |  |  |  |  |  |  |  |  |  |
| Attendant cut cord with new or sterile blade |  |  |  |  |  |  |  |  |  |  |  |  |  |  |  |
| No | ^1^ | ^1^ | ^1^ | ^1^ | ^1^ | 1.1 | 0.5 | 0.6 | 0.4 | 0.8 | ^1^ | ^1^ | ^1^ | ^1^ | ^1^ |
| Yes | ^1^ | ^1^ | ^1^ | ^1^ | ^1^ | 98.9 | 99.5 | 99.4 | 99. | 99.2 | ^1^ | ^1^ | ^1^ | ^1^ | ^1^ |
| Baby was kept warm or wrapped within 10 minutes of delivery |  |  |  |  |  |  |  |  |  |  |  |  |  |  |  |
| No | 34.5 | 54.2 | 52.7 | 52.5 | 43.4 | 33.5 | 27.1 | 29.4 | 23.3 | 30.2 | 99.7 | 99.4 | 99.6 | 99.2 | 99.6 |
| Yes | 65.5 | 45.8 | 47.3 | 47.5 | 56.6 | 66.5 | 72.9 | 70.6 | 76.7 | 69.8 | 0.3 | 0.6 | 0.4 | 0.8 | 0.4 |
| Baby was bathed within 24 hours of delivery |  |  |  |  |  |  |  |  |  |  |  |  |  |  |  |
| No | 58.8 | 61.5 | 60.7 | 66.1 | 60.1 | 37.9 | 17.5 | 19.5 | 14.2 | 27.5 | 60.4 | 51.9 | 58.1 | 47.5 | 56.6 |
| Yes | 41.2 | 38.5 | 39.3 | 33.9 | 39.9 | 62.1 | 82.5 | 80.5 | 85.8 | 72.5 | 39.6 | 48.1 | 41.9 | 52.5 | 43.4 |
| Total (home deliveries for all livebirths) | 2,723 | 2,251 | 900 | 936 | 4,559 | 6,129 | 6,422 | 3,963 | 2,459 | 12,551 | 1,515 | 1,246 | 516 | 730 | 2,761 |
| **Breastfeeding practices for all liveborn newborns** |  |  |  |  |  |  |  |  |  |  |  |  |  |  |  |
| Breastfed within 1 hour of delivery |  |  |  |  |  |  |  |  |  |  |  |  |  |  |  |
| Not breastfed within 1 hour | 28.3 | 18.4 | 21.6 | 14.4 | 23.4 | 32.1 | 20.9 | 22.5 | 18.1 | 26.4 | 33.3 | 25.1 | 25.8 | 24.7 | 29.1 |
| Breastfed within 1 hour | 71.7 | 81.6 | 78.4 | 85.6 | 76.6 | 67.9 | 79.1 | 77.5 | 81.9 | 73.6 | 66.7 | 74.9 | 74.2 | 75.3 | 70.9 |
| Exclusive breastfeeding for 6 weeks following birth |  |  |  |  |  |  |  |  |  |  |  |  |  |  |  |
| Not breastfed exclusively for 6w | 16.3 | 11.4 | 10 | 13 | 14.1 | 26.5 | 21.8 | 22.6 | 20.6 | 24.1 | 12.9 | 15.3 | 18.9 | 13.5 | 14.1 |
| Breastfed exclusively for 6w | 83.7 | 88.6 | 90 | 87 | 85.9 | 73.5 | 78.2 | 77.4 | 79.4 | 75.9 | 87.1 | 84.7 | 81.1 | 86.5 | 85.9 |
| Total (live births) | 4,906 | 4,591 | 1,805 | 1,885 | 8,596 | 8,542 | 8,776 | 5,550 | 3,216 | 17,308 | 3,439 | 3,603 | 1,211 | 2,392 | 7,042 |

^1^ Data for this outcome were not collected in this study.

^2^ Although data was collected for this study, it is included, as it was not discussed in women’s groups meetings.
